# Integrating mangrove growth and failure in coastal flood protection designs

**DOI:** 10.1038/s41598-024-58705-4

**Published:** 2024-04-04

**Authors:** A. Gijón Mancheño, V. Vuik, B. K. van Wesenbeeck, S. N. Jonkman, R. van Hespen, J. R. Moll, S. Kazi, I. Urrutia, M. van Ledden

**Affiliations:** 1 Delft University of Technology, Stevinweg 1, Delft, 2628 CN The Netherlands; 2 HKV Consultants, P.O. Box 2120, Lelystad, 8203 AC The Netherlands; 3 Department of Ecosystems and Sediment Dynamics, Deltares, P.O. Box 177, Delft, 2600 MH The Netherlands; 4 World Bank, 1818 H Street, Washington, DC 20433 USA; 5 Department of Estuarine and Delta Systems, WNIOZ Yerseke, Royal Netherlands Institute for Sea Research and Utrecht University, Utrecht, Netherlands

**Keywords:** Ecosystem services, Restoration ecology, Wetlands ecology, Civil engineering

## Abstract

Mangrove forests reduce wave attack along tropical and sub-tropical coastlines, decreasing the wave loads acting on coastal protection structures. Mangrove belts seaward of embankments can therefore lower their required height and decrease their slope protection thickness. Wave reduction by mangroves depends on tree frontal surface area and stability against storms, but both aspects are often oversimplified or neglected in coastal protection designs. Here we present a framework to evaluate how mangrove belts influence embankment designs, including mangrove growth over time and failure by overturning and trunk breakage. This methodology is applied to *Sonneratia apetala* mangroves seaward of embankments in Bangladesh, considering forest widths between 10 and 1000 m (cross-shore). For water depths of 5 m, wave reduction by mangrove forests narrower than 1 km mostly affects the slope protection and the bank erodibility, whereas the required embankment height is less influenced by mangroves. *Sonneratia apetala* trees experience a relative maximum in wave attenuation capacity at 10 years age, due to their large submerged canopy area. Once trees are more than 20 years old, their canopy is emergent, and most wave attenuation is caused by trunk and roots. Canopy emergence exposes mangroves to wind loads, which are much larger than wave loads, and can cause tree failure during cyclones. These results stress the importance of including tree surface area and stability models when predicting coastal protection by mangroves.

## Introduction

Mangrove forests are tropical and sub-tropical coastal ecosystems that reduce flood risk along coastlines worldwide^1–4^, among many other ecosystem services^5^. Wave and surge attenuation by mangroves is estimated to reduce flood protection costs by 65 billion USD per year^2^, and could decrease the adaptation costs of coastal infrastructure by 71-168 billion USD by 2080^4^. Besides those benefits, mangroves can counteract up to 7 mm/year of sea level rise by trapping sediment^6,7^ and building up peat^8^, and they sequestrate carbon and mitigate global warming^9^. Mangroves are thus being increasingly considered in coastal protection plans, and specifically in the design of coastal embankments^4,10^. Nevertheless, existing studies of flood mitigation by mangroves^2–4^ highly simplify the vegetation characteristics, for instance by neglecting vertical changes in the vegetation surface area, or assuming constant mangrove properties worldwide. This can introduce large inaccuracies in predictions of flood risk reduction by mangroves, as there are many different species across the globe, with very different geometries^11^ and present at varying tidal elevations^12–15^. Moreover, mangrove forests can be damaged during extreme events^16,17^, while existing studies of flood risk reduction by mangroves often neglect vegetation failure and its effect on coastal protection.

The aim of this paper is thus to develop a framework to quantify the effect of narrow mangrove belts (< 1 km) on embankment designs, considering how their protective capacity varies over time. The framework includes changes in tree frontal surface area with age, as well as failure mechanisms of the mangroves trees (overturning and trunk breaking). The method is applied to the coastal system of Bangladesh, since this country is very vulnerable to cyclones^18^ and is home to extensive mangrove areas^19,20^. Bangladesh is exposed to frequent cyclones, which induce water levels of up to 13 m, and has a large population living in low-lying areas^18,21^. Its coastal system is therefore defended by a system of 6000 km of peripheral embankments that enclose 139 polders. As the embankment system is being upgraded to provide sufficient safety against rising water levels, integration of mangroves into embankment designs could reduce the design requirements of the structures^2–4,22–24^, besides giving additional resilience against climate change^9^, enhancing the local biodiversity^25^, and increasing the water quality^26^ among other benefits^5^. Narrow mangrove fringes are likely to have a small effect on surge levels, since observed attenuation rates at other countries showed surge reductions of 0-0.25 m per km of forest^27–31^, and modelling studies in Bangladesh support those observations^10^. However, mangroves can significantly attenuate short waves, between 5 and 100% for a fringe of 100 m (from sea to land)^32–35^, which would reduce the wave loads on coastal embankments.

The effect of narrow mangrove fringes on waves is here quantified with a wave attenuation model based on that of Mendez and Losada^36^ (see “Wave model” section). The frontal surface area of *Sonneratia apetala* trees is obtained from the analysis of field pictures, as shown in the “Methods” section, while changes in vegetation surface area over time are modelled using empirical expressions for tree growth from the literature^37,38^. The potential failure of the trees by either overturning or trunk breakage is estimated by comparing the moment due to the forces induced by wind and waves with the resisting moment of the vegetation, as explained in the “Mangrove failure” section. Finally, the calculated wave heights are used as an input in the design formulas of embankments^39–41^ to evaluate how the presence of a mangrove belt would influence their height, slope protection needs, and the wave-driven shear stresses at their toe (see section on “Effect of mangroves on embankment design”).

## Theoretical background

### Mangrove geometry

Wave attenuation by a mangrove forest varies with tree geometry and relative ground elevation with respect to the tidal levels. Mangroves inhabit sheltered coastal areas, above mean sea level and below mean high water, where different species usually distribute along shore-parallel bands depending on their propagule dispersal^42^ and their ability to survive the thresholds to early establishment^43^. In Bangladesh *Avicennia officinalis* (Baen) and *Sonneratia apetala* (Keora) are pioneer species at exposed coastal sites, which have been extensively planted along the coastal system due to their high survival rates compared to other mangrove species^19^.

**Figure 1:**
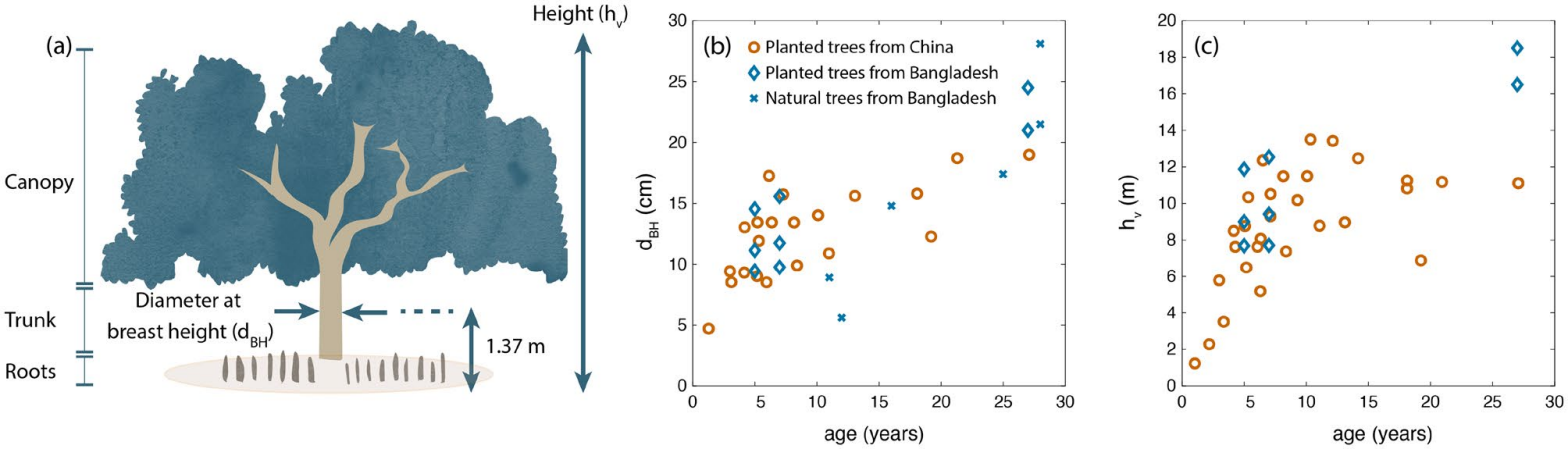
(**a**) Parts of a *Sonneratia apetala* mangrove, including canopy (formed by leaves and branches), trunk, and pneumatophore roots. (**b**, **c**) *S. apetala* size as a function of tree age for natural forest stands in Bangladesh^46^ and for planted trees in Bangladesh^37,38^ and China^48^. Plot (**b**) presents the trunk growth, represented by the diameter at breast height $d_{BH}$ (cm), and plot (**c**) displays changes in tree height $h_v$ (m), from Gijón Mancheño et al.^50^.

When mangroves colonize a new site, their wave reduction efficiency rises as they grow their trunk^44^, and develop their root^44^ and branch system. Their growth rate is influenced by multiple biophysical parameters, like soil composition^45^, water salinity^46^, or temperature^47^. Differences in growth between sites are illustrated in Fig. 1 for *S. apetala* mangroves in Bangladesh^37,38^ and China^48^. Mangrove plantations of less than 20 years old show similar trunk diameters in both countries (Fig. 1b, c), but for older ages, mangroves in Bangladesh grow taller at the sites compared in Fig. 1b, c. The larger size of trees in Bangladesh could be caused by a more suitable habitat (e.g., a more favourable climate), given that *S. apetala* is native of the Sundarbans and was imported into China during the 80s^49^, but local differences between sites could also explain variations in growth.

**Table 1:** *S. apetala* mangrove characteristics from field studies, based on Gijón Mancheño et al.^50^.

Author	Country	$h_{r}$ [cm]	$d_{r}$ [cm]	$N_{r}$ [roots/m^2^]	$d_{BH}$ [cm]	$h_v$ [m]	$N_v$ [trees/m^2^]	Age [year]
Chen et al. (2021)	China	8	–	–	7	2.3	0.01	2
Duan et al. (2021)	China	18	0.8	–	35	14.8	0.17	–
Zhang et al. (2019)	China	12	–	253	–	18.3	0.17	–
Mazda et al. (2005)	Vietnam	14	0.7	131	12	–	–	
Dasgupta et al. (2017)	Bangladesh	104	15	–	51	–	0.01–0.04	–

The table lists study authors, country, and mangrove tree characteristics: pneumatophore height ($h_r$, in cm), pneumatophore diameter ($d_r$, in cm) pneumatophore density ($N_r$, pneumatophores per m^2^), mangrove trunk diameter at breast height $d_{BH}$ (cm), mangrove height ($h_v$, in m), mangrove density ($N_v$, mangroves per m^2^), and mangrove age in years.

Although the data in Fig. 1 provides an indication of how the trunk diameter and tree height change as *S. apetala* trees grow, while Table 1 gives an impression of the relationship between root characteristics and tree height, the evolution of the total frontal area of *S. apetala* mangroves with age has not been measured in the field. For other mangrove species, the equivalent canopy width has been defined using several approaches, like assuming that the width of the canopy is given by the outer contour of the tree crown including^51^ or neglecting^32,33^ voids and empty areas. Other studies express the canopy area as a field of cylinders^52^, but without basing the canopy area on field measurements of the total branch area. Lastly, at sites where the canopy is emergent, the tree area above the root system is given by the area of the trunk^53^.

When mangrove trees are fully submerged, using the full canopy volume in wave calculations would overestimate the obstruction exerted by the trees, as part of the canopy is porous to the flow. Since leaves were found to have a small effect on wave attenuation for willow trees due to their high flexibility^54^, and they can also be pulled by wind and waves^55^, including the full leaf area in wave reduction predictions most likely overpredicts the effect of trees during extreme events. For willow trees, tree trunk and branch models were developed by Kalloe et al.^56^ and successfully used to predict wave attenuation measurements by van Wesenbeeck et al.^54^ Models for the growth of the trunk and root system of *Rhizophora sp.* have also been developed by Maza et al.^44^ However, geometry models are not available for the pioneer species in Bangladesh.

### Mangrove stability against storms

Mangroves can protect the coast from storm events but they can also be degraded by the impact of winds and waves. Hurricanes and cyclones can directly damage mangroves by defoliating them, by breaking their trunk or branches, or by overturning them^57–59^. Extreme events can also damage mangroves by altering their habitat, for instance, by changing the local salinity, hydrology, topography, sediment conditions, or the forest structure^60^. In Bangladesh the cyclone Sidr (2007) damaged between 11%^16^ and 45%^17^ of the total area of the Sundarbans. Large-scale cyclone damage in the Sundarbans (where more than 5% of the forest is affected) is estimated for wind speeds exceeding 100 km/h^61^, based on observations of past cyclones. This order of magnitude is also consistent with records of forest damage in Florida^58^, which indicated that few trees are broken or turned over for wind speeds between 119 and 153 km/h. However, the cyclone Reshmi (with maximum 1-min sustained wind speeds of 85 km/h) locally caused structural damages over > 60% at coastal areas along the cyclone eyepath, even though the total damage at the scale of the Sundarbans was small^59,62^. Mangroves can recover over time^16^ but landward areas may be vulnerable to storm damages while the vegetation is recuperating^63^. Predicting mangrove degradation due to cyclones is thus necessary to assess the long-term forest resilience, but this step is hindered by the absence of mangrove stability models.

Trunk breakage and tree overturning have been investigated for pines, cedars, and other terrestrial trees under the action of wind loads^64^. When mangroves are submerged during storms, wave forces could also destabilize trees. The importance of waves versus wind is probably a function of tree submergence, with wave loads being more dominant for rising water levels. Younger (and shorter) trees will thus be relatively more exposed to waves whereas older (and taller) trees may be more exposed to winds. Previous studies have investigated the stability thresholds for mangrove seedlings^43^, studied the strength of mangrove branches of different species^65^, and compiled observations of mangrove failure during hurricanes^58,59,61,62,66^, but predictive models for mangrove stability against winds and waves are still lacking.

## Model development

**Figure 2:**
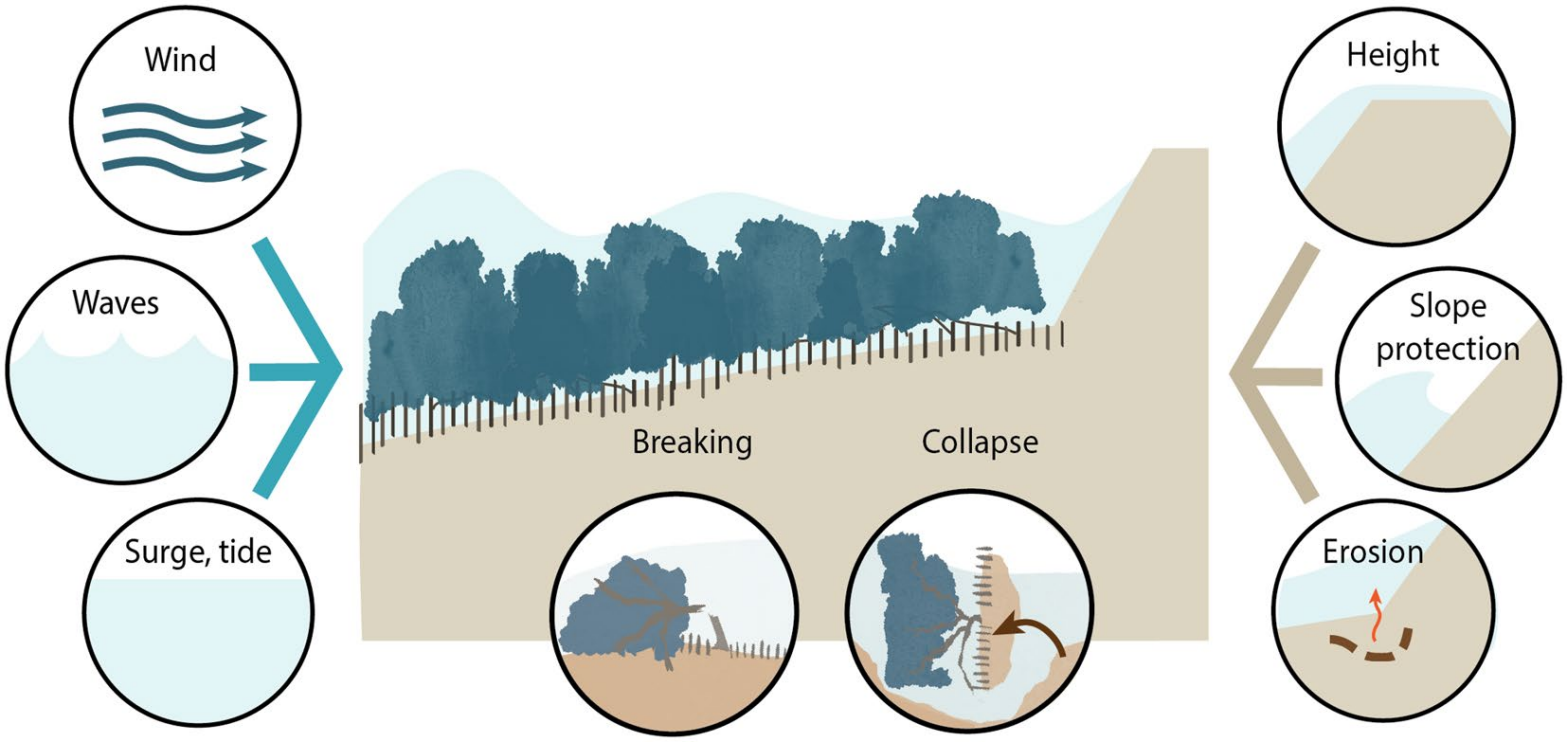
Illustration of the method to integrate mangroves in the design of coastal embankments, modified from Gijón Mancheño et al.^50^. This methodology has several steps: (1) reducing wave attack due to the presence of a mangrove belt as a function of the local surge level and wave height, (2) assessment of tree stability against waves and winds, and (3) calculation of embankment design (considering wave attenuation by the trees), specifically the structure crest height, size of slope revetment blocks, and erodibility of the embankment toe.

The methodology to incorporate a mangrove belt in the design of a coastal embankment is illustrated in Fig. 2. This method comprises: (1) a wave propagation model, (2) an assessment of potential vegetation failure due to wave and wind loads, and (3) the calculation of the embankment design.

### Wave transformation

#### Wave model

Cross-shore wave transformation is computed from the wave energy balance^36^:

1 $$\begin{aligned} \ \frac{\partial E c_g \cos {\theta }}{\partial x} = - \varepsilon _b -  \varepsilon _v \ \end{aligned}$$

where *E* is the wave energy per unit area (J/m^2^), $c_g$ is the group celerity (m/s), $\theta$ is the mean wave direction (rad), $\varepsilon _b$ represents wave dissipation due to depth-induced breaking (W/m^2^), and $\varepsilon _v$ represents wave dissipation by mangrove trees (W/m^2^). Here we have assumed that wave dissipation by bed friction is an order of magnitude smaller than the energy loss due to wave breaking or due to wave dissipation by the vegetation^36^. Equation 1 is solved using a forward stepping scheme from the offshore boundary towards the land over cells of size $\Delta x$ and constant water depth, as done by Mendez and Losada^36^.

Wave dissipation due to depth-induced breaking is computed using the formulation of Thornton and Guza^67^:

2 $$\begin{aligned} \  \varepsilon _b = \frac{3 \sqrt{\pi }}{16} \rho _w g \frac{B^3 f_p}{\gamma _{br}^4 h^5} H_{rms}^7 \ \end{aligned}$$

Where $f_p$ is the peak frequency (s^-1^), $\rho _w$ is the water density (kg/m^3^), *g* is the acceleration of gravity (m/s^2^), $H_{rms}$ is the root-mean-square wave height (m), and *B* (-) and $\gamma _{br}$ (-) are empirical coefficients that are set to the default values used by Mendez and Losada^36^: $B = 1$ and $\gamma _{br} = 0.6$.

Wave dissipation by the mangrove vegetation is modelled as the work of the hydrodynamic forces acting on the trees. The wave load acting on each tree is estimated using the formulation for hydrodynamic forces acting on cylinders of Morison et al.^68^:

3 $$\begin{aligned} \ F_w = \int _{-h}^{-h+h_v} \left( \frac{1}{2}\rho _w c_{D,w} d u_w(z)|u_w(z)|+ \rho c_M \frac{\pi d^2}{4} \frac{\partial u_w}{\partial t}\right) \partial z,\ \end{aligned}$$

where $c_{D,w}$ and $c_M$ are the bulk drag and inertia coefficients under waves (-), *d* is the diameter of the cylinder (m), which represents individual roots, trunk, and branches, $u_w(z)$ is the orbital velocity associated to the root-mean-square wave height (m/s), *z* is the height from the ground (m), *h* is the water depth (m), and $h_v$ is the tree height (m). Large-scale experiments with willow trees provided values of $c_{D,w} = 0.7-2$. For circular cylinders, $c_M = 0-2$^68^.

The depth-integrated and time-averaged work over a forest is calculated as:

4 $$\begin{aligned} \  \varepsilon _v = \frac{1}{T_p} \overline{\int _{-h}^{-h+h_v} \frac{1}{2}\rho _w c_{D,w} b_v(z) N_v u_w(z)^3 dz dt} \ \ \end{aligned}$$

where $T_p$ is the peak wave period (s), *t* is the time (s), $b_v$ is the integrated tree width at an elevation *z* from the ground (m), which corresponds with the sum of the widths of all branches or roots at a given height *z*, and $N_v$ is the tree density per unit area (trees/m^2^). Equation (4) is integrated numerically over the vertical coordinate over cells with a height $\Delta z$ and varying width $b_v(z)$ and wave orbital velocity $u_w(z)$.

#### Validation of wave model

The model predictions of wave attenuation are validated for several scenarios: (1) an unvegetated profile, (2) a vegetated profile where plant properties are constant over the vertical but vary along the cross-shore profile, and (3) a vegetated profile where plant properties vary over the vertical but remain constant along the profile. These different cases are modelled to ensure that the modules of wave breaking (Eq. 2) and wave dissipation by vegetation (Eq. 4) are well implemented, and to validate both the horizontal (Eq. 1) and vertical (Eq. 4) integration of the energy dissipation terms.

**Figure 3:**
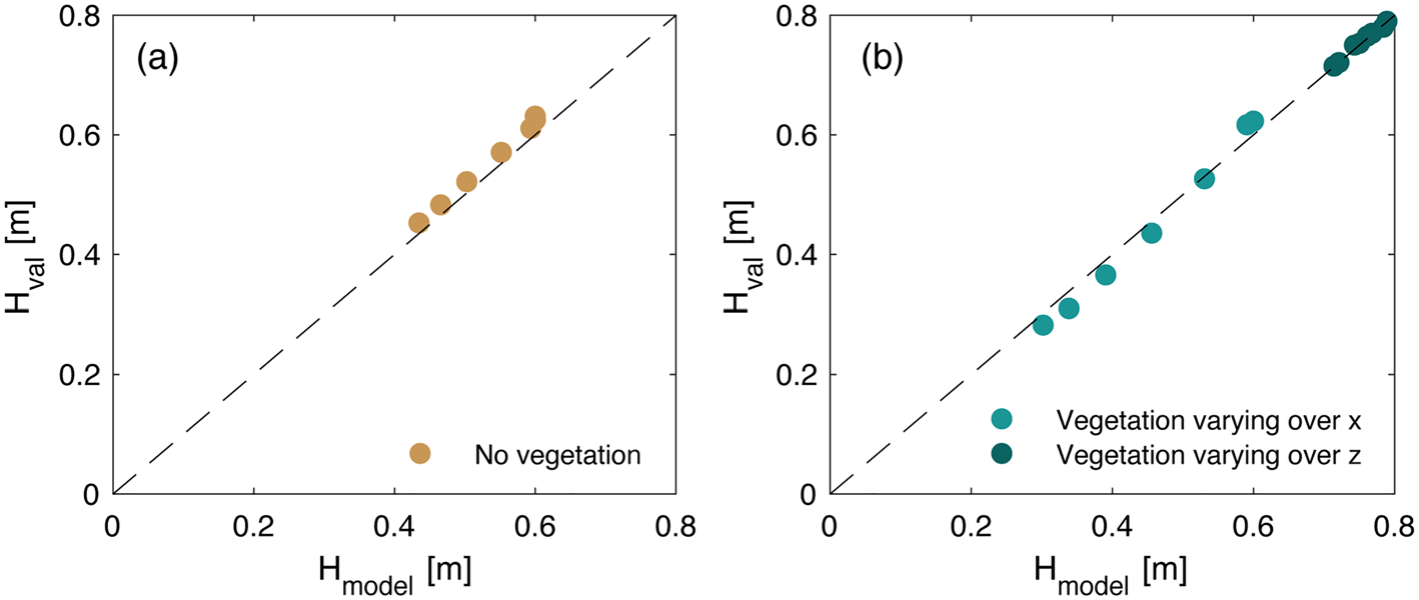
Comparison between wave heights obtained using the wave model given by Eqs. (1–4), $H_{model}$, and the validation wave heights $H_{val}$ from (**a**) model results obtained with an unvegetated profile by Vuik et al.^22^, and (**b**) case studies with vegetation from Wesenbeeck et al.^54^ (dark green dots) and Vuik et al.^22^ (light green dots).

The first two situations are tested against the results of Vuik et al.^22^, who measured wave propagation through a salt marsh fringe and modelled it using SWAN. Wave transformation through the salt marshes is modelled for a significant wave height of $H_{m0} = 0.6$ m and a peak period of $T_p = 3.5$ s at the offshore boundary. The bathymetry varies between an offshore water depth of 2 m, and a nearshore water depth of 0.5 m. The salt marshes extend over a (cross-shore) width of 55 m and have uniform properties over the vertical coordinate and varying vegetation properties across the profile, with $h_v$ = 0.2–0.3 m, $b_v$ = 2.7–3 mm and $N_v$ = 944–1520 plants/m^2^. A drag coefficient of $c_{D,w} = 0.4$ is used in the simulations, which is equal to the value calibrated by Vuik et al.^22^ The model output shows good agreement with the results of Vuik et al.^22^ at all cross-shore locations, with and without vegetation (Fig. 3).

In the third situation, with vegetation properties changing over the vertical, the model is tested for the conditions of the large-scale flume experiments with pollarded willows of van Wesenbeeck et al.^54^, where waves travelled across 32 willow trees with a height of 6 m, and a total forest width of 40 m. This study is selected as it is the only large-scale wave experiment with real trees during extreme conditions that provides high-resolution information of the tree canopy, measured with terrestrial laser scanners. Offshore wave heights (at the wave maker), wave periods, and water depths (at the location of the trees) reached values of $H_{m0} = 0.4-1.4$ m, $T_p = 2.8-6.7$ s, and $h = 3-4.5$, respectively. The vertical distribution of the tree surface area was quantified by Kalloe et al.^56^, who measured vegetation parameters varying between $b_v$ = 0.03–0.3 m over the tree height, for trees placed with a density of $N_v$ = 1 tree/m^2^. The input drag coefficients for the validation cases were obtained from van Wesenbeeck et al.^54^ and range between $c_{D,w} = 1.02-1.87$. The modelled wave decay through the forest also agrees with the measurements by van Wesenbeeck et al.^54^, as illustrated in Fig. 3b.

### Mangrove failure

#### Wind loads

The wind forces acting on a mangrove tree are estimated with a quadratic (form) drag equation:

5 $$\begin{aligned} \ F_{a} = \int _{0}^{-h+h_v} \frac{1}{2} \rho _{a} c_{D,c} b_v(z) G u_a(z)^2 dz \ \end{aligned}$$

Where $\rho _a$ is the air density (kg/m^3^), $c_{D,c}$ (-) is the drag coefficient for wind flows, which varied between 0.2 and 1 in wind tunnel experiments with terrestrial trees^69^, with lower $c_{D,c}$ values corresponding with higher tree deflection and higher values representing small tree motion, *G* is an empirical gust factor that is given a value of 1.2 for high wind speeds^70^ (-), and $u_a$ is the maximum sustained wind speed relative to the tree motion (m/s), measured at 10 m from the ground and averaged over a period of 1–3 min. Above the vegetation, the wind speed is often assumed to follow a logarithmic velocity profile over the vertical coordinate *z*^64^:

6 $$\begin{aligned} \ u_a (z) = \frac{u_a^*}{k} ln \frac{z}{z_0} \end{aligned}$$

Where $z_0$ is the roughness height (m), which can reach values of the order of 0.0002 m for water, 0.02 m for unvegetated sites^70^, and up to 5 m for tall forests^64^, $u_a^*$ is the friction velocity (m/s), and *k* is the von Karman constant (-), equal to 0.4.

#### Moment caused by waves and wind

For an emergent tree situation, the potential for tree failure is calculated using the overturning moment acting on the tree:

7 $$\begin{aligned} \ M = \int _{-h}^{0} dF_w(z) z dz + \int _{0}^{-h+h_v} dF_a(z) z dz \ \end{aligned}$$

where the overturning moment is generated by the wind-driven force (Eq. 5) above the water surface, and by the wave-driven force (Eq. 3) below water. For a fully submerged tree the wind component would be equal to zero, and the overturning moment due to waves would be integrated from $z = -h$ to $z = -h+h_v$.

#### Resisting moment to breakage

Trunk breakage is estimated by comparing the overturning moment experienced by the tree *M* (Eq. 7) with the maximum bending moment that a tree can withstand without breaking, $M_{break}$^71^:

8 $$\begin{aligned} \ M_{break} = \frac{\pi }{32} f_{knot} \sigma _u d_{BH}^3 \ \end{aligned}$$

Where $\sigma _u$ is the modulus of rupture of the wood tissue (N/m^2^), $d_{BH}$ is the trunk diameter at breast height (m), and $f_{knot}$ is a factor that reduces the wood strength due to the presence of knots (-), usually between $f_{knot} = 0.8-1$.

#### Resisting moment to toppling over

Tree failure by toppling over can be estimated by comparing the bending moment acting on the tree with the critical overturning moment $M_{over}$, measured in tree pulling experiments^72^:

9 $$\begin{aligned} \ M_{over} = C_r W \ \end{aligned}$$

Where $C_r$ is a dimensional regression constant varying between $C_r= 60-200$ m^2^/s^2^, and *W* is the tree trunk weight in kg from empirical relationships derived from field measurements.

#### Evaluation of mangrove failure model

To evaluate the model predictions of mangrove stability (Eqs. 7–9), since we could not find observational studies of *S. apetala* tree failure that provided all required modelling variables at one site (e.g., local wind speed, water level, vegetation damage, vegetation properties and soil characteristics), we have conducted a semi-quantitative verification of the tree failure model gathering information from several studies to define the necessary modelling parameters.

The case studies used to evaluate the tree failure model for *S. apetala* are the cyclone Sidr (2007) in Bangladesh, and the thyphoon Mangkhut (2018) in China. In the Sundarbans (Bangladesh), *S. apetala* trees with heights above 15 m were broken during cyclone Sidr (2007)^73^. The location of the damaged trees was not specified, but mangrove species maps show that *S. apetala* trees are mostly present along the south east of the Sundarbans^74^, where water depths varied between 2 and 6 m during Sidr (2007)^75,76^, and where 1-min sustained wind speeds varied between 60-180 km/h^77^. In Honghai wan (China), 6-m tall *S. apetala* planted mangroves were able to stay intact during thyphoon Mangkhut (2018) with water depths up to 3 m in the mangrove forest^78^. The typhoon landed 200 km away from the mangrove site with maximum 2-minute sustained winds of 160 km/h, whereas at the nearby city of Shanwei (13 km away from the mangrove plantation), fallen trees, traffic accidents, and damaged advertisement boards were reported^78^. Such wind damages approximately correspond with Beaufort scales 9-10, and with wind speeds between 75 and 102 km/h.

To estimate the forces acting on the trees during cyclone Sidr (2007) and thyphoon Mangkhut (2018), we assume depth-limited waves in shallow water, and that tree swaying takes place during cyclonic conditions (and thus $c_{D,w} = 0.7$, based on large-scale wave experiments with natural willow trees, and $c_{D,c} = 0.2$ based on wind tunnel experiments with western red cedars^69^). The forces are calculated at the edge of the forest (neglecting canopy effects), and we assume $c_M = 2$ and $f_{knot} = 1$ in tree stability calculations. The surface area of *S. apetala* trees is estimated using the model of the “Modelling scenarios” section (shown in Fig. 5b) and their tree weight using the biomass allometric relationships of Zhu et al.^79^ Mangrove stability is calculated using the full range for the empirical coefficient for overturning ($C_r= 60-200$ m^2^/s^2^^72^), since there is no specific information of $C_r$ values for mangroves, and using the expected range of modulus of rupture for *S. apetala* ($\sigma _u$ = 37±7 N/mm^2^)^55^.

**Figure 4:**
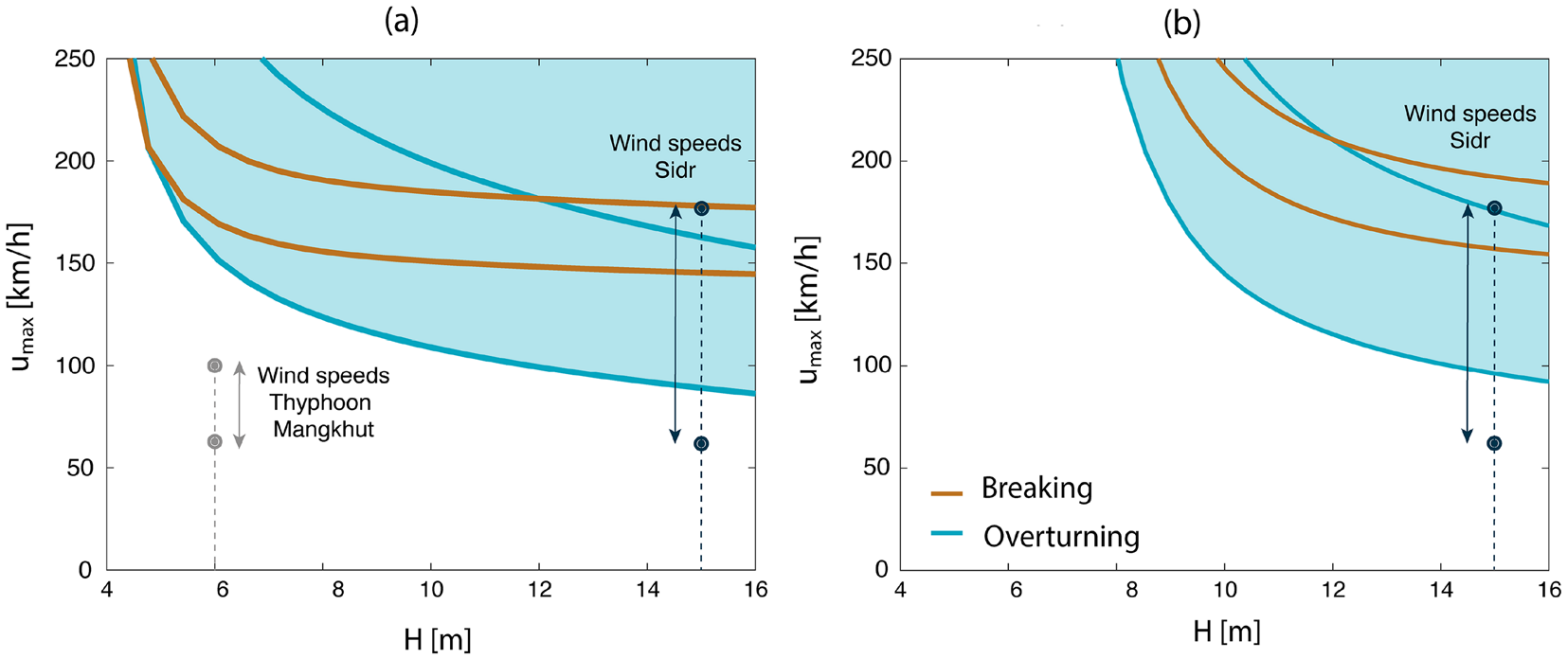
Maximum wind speed that mangroves can withstand without failing ($u_{max}$) as a function of tree height (*H*). The white region of the plots corresponds with combinations of wind speed and tree height for which there is no failure, whereas the areas filled in blue are the combinations for which failure occurs. The maximum wind speed for overturning is shown by blue lines, and the maximum wind speed for trunk breaking by brown lines. The lower and upper blue lines correspond with the minimum and maximum value of the empirical coefficient for overturning for terrestrial trees^72^, and for the brown lines with the minimum and maximum value for the modulus of rupture of *S. apetala* trees^55^. Plot (**a**) shows model results with a total water depth of 3 m, and (**b**) shows results obtained with a total water depth of 6 m.

For the conditions expected during Thyphoon Mangkhut, model results show no damage for tree heights of 6 m (Fig. 4a). The planted mangroves did not topple over, probably because they were part of a 6-year-old plantation with relatively short trees (Fig. 1). Failure could have occurred to trees with more than 10 m in height, or for 6-m tall trees if the cyclone had been closer to the mangrove forest and caused stronger local winds. It should be noted that the sustained wind speeds of Fig. 4 are multiplied by a gust factor to estimate the drag forces (factor *G* in Eq. 5), since gusts are large determinants of tree failure^80^.

The local water depth was not reported at mangrove sites damaged by Sidr (2007), and we therefore model two possible water depths (Fig. 4a, b), to evaluate what would happen for the expected range of maximum wind speeds. Model results support that the relative timing between surge level and wind speed largely influences mangrove failure. High water levels shelter mangroves from the wind, specially for young individuals. For instance, 15-m tall *S. apetala* trees would fail under winds exceeding 90 km/h with a surge level of 3 m (Fig. 4, a), but could withstand stronger winds, of 96 km/h, with a water depth of 6 m (Fig. 4, b). Model results thus agree with the observation that failure can occur for 15-m tall trees under most of the wind range experienced during Sidr (2007), however, more detailed measurements are recommended for a precise model validation, and to narrow down the range of $C_r$ for mangroves.

### Effect of mangroves on embankment design

#### Embankment height

The height of an embankment is usually chosen in such a way that the overtopping discharge remains below a critical threshold, which depends on the erosion resistance of the crest and inner slope of the embankment. Mangroves reduce the value of the significant wave height $H_{m0}$ at the structure (where $H_{m0} = \sqrt{2} H_{rms})$, and thus decrease the overtopping discharge for a given design. The expected value of the overtopping discharge over a sloping embankment *q* can be calculated using the formula of EurOtop^39^:

10 $$\begin{aligned} \ q = \sqrt{g H_{m0}^3} \frac{f_1}{tan(\alpha )} \gamma _b  \varepsilon _{m-1.0} e^{- \left( f_2 \frac{h_{crest}-h}{ \varepsilon _{m-1.0} H_{m0} \gamma _b \gamma _f \gamma _{\beta } \gamma _{\nu }}\right) ^{f_3}} \ \ \end{aligned}$$

where *q* is the overtopping discharge per meter (m^3^/m/s), $f_1$ is an empirical factor equal to 0.026 (-), $\alpha$ is the angle of the outer slope (-), $\varepsilon _{m-1.0}$ is the breaker parameter (-), $\gamma _b$ is the influence factor for a berm (-), $\gamma _f$ is the influence factor for roughness elements on the slope (-), $\gamma _\beta$ is the influence factor for oblique wave attack (-), $\gamma _\nu$ is the influence factor for vertical wall (-), $f_2$ is an empirical factor equal to 2.5 (-), $h_{crest}$ is the crest level (m), *h* is the water depth at the embankment toe (m), and $f_3$ is an empirical factor equal to 1.3 (-).

#### Slope protection

Wave attenuation by mangroves can also reduce the stone weight required to protect the embankment slope. The slope protection of the embankment can be calculated with the expression of Pilarczyk^40,41^.

11 $$\begin{aligned} \ \frac{H_{m0}}{\Delta D}= \frac{F \cos {\alpha }}{ \varepsilon _{m-1.0}^b} \ \end{aligned}$$

Where $H_{m0}$ is the spectral significant wave height at the toe of the structure (m), *D* is the thickness of the cover layer (m), $\Delta$ is the relative density of concrete with respect to water (-), *F* is a stability factor between 3 and 6 for a revetment formed by concrete blocks (-), and *b* is an exponent equal to 0.67 for semi-permeable block revetments (-).

#### Erosion at the toe

Wave attenuation by mangroves reduces the shear stresses acting at the toe of the embankment, and hence decreases the erosion rates. The effect of wave attenuation by mangroves on the shear stresses ($\tau _{b,w}$) acting on the embankment toe can be calculated according to Equation 12:

12 $$\begin{aligned} \ \tau _{b,w} = \frac{1}{4} \rho _w f_w u_{w,b}|u_{w,b}|\ \end{aligned}$$

where $u_{w,b}$ is the orbital velocity (m/s) associated to the root-mean-square wave height at the sea bottom ($z = - h$), $f_w$ is the wave friction factor (-), defined as^81^:

13 $$\begin{aligned} \ f_w = min \left( \exp {\left( -6+5.2\left( \frac{u_{w,b}}{2.5 d_{n50} \omega _m}\right) ^{-0.19} \right) },0.3 \right) \ \end{aligned}$$

with $d_{n50}$ being the mean grain size (m) and $\omega _m$ the mean wave frequency (rad/s).

### Modelling scenarios

#### Tree geometry model

In order to evaluate the effect of *S. apetala* mangroves over time, a model of the tree surface area is developed using field pictures and literature data. For a given tree age, the height $h_v$ and trunk diameter $d_{BH}$ are obtained from logarithmic fits to the data of *S. apetala* trees from Bangladesh (shown by blue dots in Fig. 1). Equation 14 provides the fitted tree height (m) as a function of age (years):

14 $$\begin{aligned} \ h_v = 9.72 \log {(age + 2.953)} - 10.3\ \end{aligned}$$

The tree height is capped to a maximum value of 18 m, as it is the maximum *S. apetala* height found in the literature, which also agrees with the mean value of *S. apetala* tree height observed by Rahman et al.^25^ at selected plots in the Sundarbans. The diameter at breast height (cm) as a function of age (years) is obtained from Eq. (15):

15 $$\begin{aligned} \ d_{BH} = 7.07 \log {(age + 2.953)} - 6.55\ \end{aligned}$$

**Figure 5:**
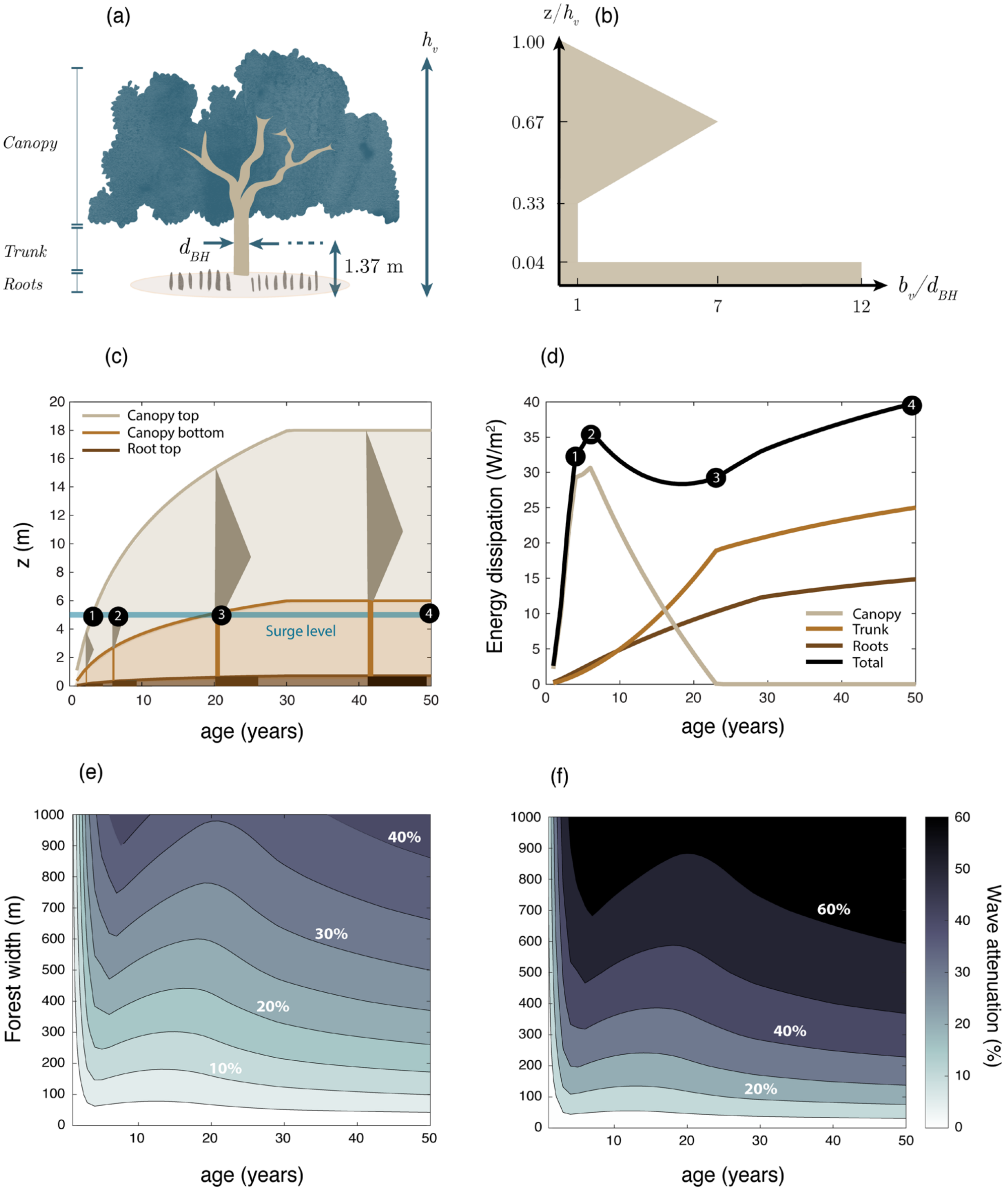
(**a**) Diagram illustrating the different sections of a tree: canopy, trunk, and roots. (**b**) The tree surface area of each section is parameterized as a function of the diameter at breast height ($$d_{BH}$$) and the tree height ($$h_v$$). The height of the different tree sections (calculated with the parameterizations of plot b) is shown as a function of tree age in (**c**), and the corresponding wave energy dissipation produced by mangroves at the seaward edge of a mangrove belt ($$D_v$$) in (**d**). The wave energy dissipation is calculated for an offshore wave height of $$H_{m0} =$$ 2 m, and a water level of $$h =$$ 5 m. The drag coefficient and the tree density are set to $$c_{D,w} =$$ 2 and $$N_v = $$ 0.1 trees/m^2^, respectively. The wave attenuation for the same conditions but varying forest widths is shown in (**e**) for $$c_{D,w} =$$ 0.7 and in (**f**) for $$c_{D,w} =$$ 2.

To define the surface area of a mangrove tree for every age, three sections are considered: roots, trunk, and canopy (Fig. 5a). The characteristics of each layer are related to the height $h_v$ and trunk diameter $d_{BH}$ by combining information from the literature (Table 1) and from the analysis of field pictures (see Fig. 9 in the “Methods” section). We assume that the ratios between layers remain the same as trees grow, and the simulations start with a 1-year old sapling, increasing the age in 1-year intervals.

The root layer is assumed to consist of cylindrical pneumatophores of equal height and width. The height of the pneumatophores is defined as 4% of the tree height ($h_{r}$ = 0.04 $h_v$), and the total root width (adding the width of all the pneumatophores), is set equal to 12 times the diameter at breast height ($b_{r} = 12$ $d_{BH}$), based on Table 1. The trunk is assumed to have a constant width equal to the diameter at breast height $d_{BH}$.

**Figure 6:**
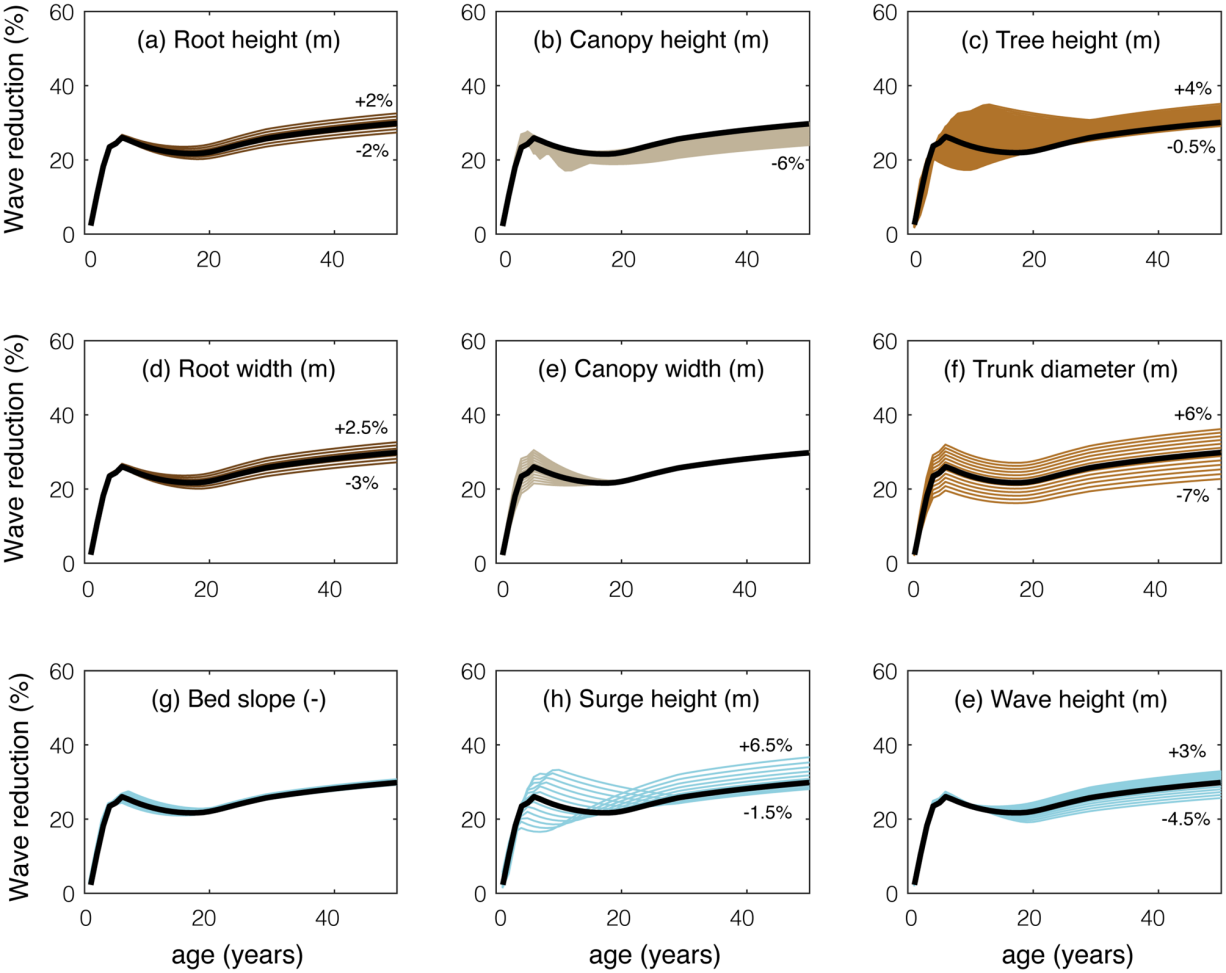
Sensitivity of wave attenuation to varying forest and hydrodynamic properties with respect to a reference scenario. The reference case is calculated with the geometrical tree model presented in Fig. 5, $c_{D,w} =$ 2.0, $N_v =$ 0.1 trees/m^2^, a forest width of 500 m, a slope of 1/1000, $H_{m0} =$ 2 m and $h =$ 5 m. The subplots show the variations in wave attenuation for changes between − 30% and + 30% of: (**a**) root height, (**b**) canopy height, (**c**) tree height, (**d**) root width, (**e**) canopy width, (**f**) trunk diameter at breast height, (**g**) bed slope, (**h**) surge height, (**i**) wave height.

The canopy characteristics are derived from the analysis of field images collected in China, shown in Fig. 9 of the “Methods” section. For the analyzed trees, the canopy started between 10 and 29 % from the ground. Considering the perspective of the field images, this height is probably underpredicted. We therefore assume that the canopy starts at 33% of the tree height ($h_{c} =$ 0.33 $h_v$) to be on the conservative side, since a lower canopy would result in a larger submerged biomass, more wave attenuation, and a stronger effect of mangroves on the embankment designs. The effect of changing the canopy height is explored in the sensitivity analysis (see Fig. 6b). The field images show that the canopy of the analyzed trees had a value of $A_{c} \approx 3 d_{BH} h_v$. As shown in Fig. 9, the surface area of the canopy increases from its base until reaching a maximum near the middle, and then decreases until the top of the tree. We schematize this vertical distribution assuming a triangular canopy with an area equal to that of the field images ($A_{c}$), which would correspond with a triangle with maximum width of $b_{v,max} = 7 d_{BH}$. The resulting tree structure as a function of the distance from the ground is shown in Fig. 5b. The evolution of the tree structure as a function of age is shown in Fig. 5c. The model sensitivity to variations of these geometrical ratios is evaluated in the “Results” section (Fig. 6a–f). The vegetation density is set to $N_v = 0.1$ trees/m^2^ (intermediate value in Table 1). The parameters of the mangrove tree geometry model are listed in Table 3 of the Supplementary material.

#### Hydrodynamic parameters

**Figure 7:**
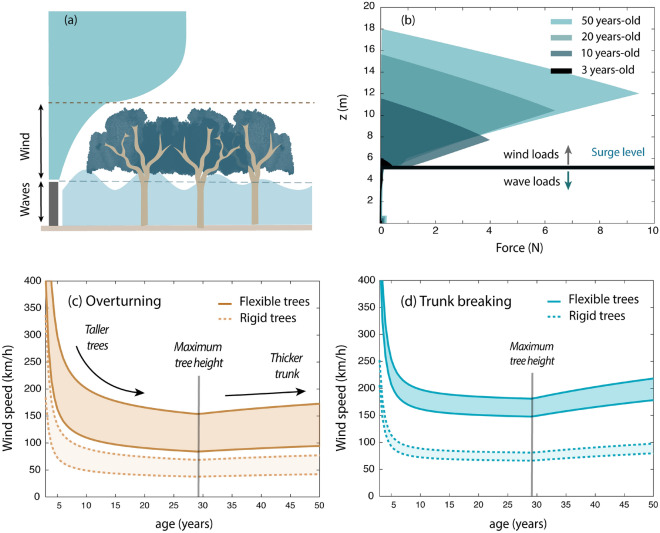
(**a**) Modelled loads on mangrove trees, consisting of wave loads (on the submerged area) and wind loads (on the emerged area). (**b**) Modelled wind forces acting on a mangrove tree placed at the seaward edge of a mangrove belt during a cyclone with wind speeds of 120 km/h. In the model, $c_{M}$ = 1, $c_{D,w}$ = 2, $c_{D,c}$ = 1, $N_v$ = 0.1 trees/m^2^, slope = 1/1000, $H_{m0}$ = 2 m and *h* = 5.5 m. (**c**) Maximum wind speed for overturning as a function of tree age. The results obtained with $c_{D,c}$ = 0.2, representing reconfiguration by flexible trees, are shown with solid brown lines, and with $c_{D,c}$ = 1, representing rigid trees, in dashed brown lines. The upper and lower lines represent minimum and maximum value of the empirical overturning parameter $C_r$ for terrestrial trees^72^. (**d**) Maximum wind speed for trunk breaking as a function of tree age. The results obtained with $c_{D,c}$ = 0.2, representing flexible trees, are shown with solid blue lines, and with $c_{D,c}$ = 1, representing rigid trees, in dashed blue lines. The upper and lower lines represent maximum and minimum value of the modulus of rupture of *S. apetala* trees^55^.

The design water depths and wave heights at the potential afforestation sites (see Figure 7 of Gijon Mancheno et al.^20^) are $$h = $$ 4.5–5 m including tides and surges and $$H_{m0} = $$ 2–3 m^82^, respectively, corresponding with a 25-year safety standard, including + 1 m in the water levels due to sea level rise (equivalent to a worst case scenario for 2050 and an average case for 2100). The report of IWM^82^ does not specify the wave period associated to the wave height but assumes a wave steepness of $$s_0 = 0.05$$, where the steepness $$s_0$$ is defined as the ratio between the wave height $$H_{m0}$$ and the wavelength *L*. We also use this steepness for the wave attenuation calculations, as we aim to test the effect of mangroves on the design condition of the structures. The corresponding wavelength is obtained as $$L = H_{m0}/s_0$$, and the wave period is derived using the dispersion relation for linear wave theory with the design water level. For the bathymetry, schematized linear profiles with slopes between 0.001 and 0.1 are considered, and the embankment toe is set at an elevation equal to mean high water (MHW).

Wave attenuation across the profile is modelled for forest (cross-shore) widths between 10 and 1000 m (seaward of the MHW point), and forest ages between 1 and 50 years old. Equations (1–4) are solved with a cross-shore grid size of $\Delta x$ = 0.01 m and a vertical grid size of $\Delta z$ = 0.005 m. For the wave loads, we assume $\rho _w$ = 1030 kg/m^3^, the drag coefficient is varied between $c_{D,w}$ = 0.7–2^54^, and the inertia coefficient is given a value of $c_{M}$ = 2^68^. For the wind loads, we assume that the mangroves are directly exposed to onshore winds (without canopy effects), $\rho _a$ = 1.2 kg/m^3^, the drag coefficient is varied between $c_{D,c}$ = 0.2–1^69^, and the roughness height is set to $z_0$ = 0.0002 m^70^ since we assess tree stability at the seaward edge of the forest. The hydrodynamic model parameters are listed in Table 1 and Table 2 of the Supplementary material.

#### Mangrove failure parameters

For the tree resistance against trunk breakage, the modulus of rupture of the wood tissue is varied within the range of $\sigma _u$ = 37 ± 7 N/mm^2^ measured by van Hespen et al.^55^, and $f_{knot}$ = 1. For overturning calculations, the tree weight is estimated using the allometric relationships of Zhu et al.^79^ and the regression constant $C_r$ is varied between $C_r= 60-200$ m^2^/s^2^^72^. The parameters of the mangrove failure model are listed in Table 4 of the Supplementary material.

#### Embankment design parameters

For the design of coastal embankments, we assume slopes of 1:8, armor layers (corresponding with $\gamma _f = 0.55$), and a berm (with $\gamma _b = 0.89$). We also assume perpendicular wave incidence (so $\gamma _\beta = 1$), and no vertical walls ($\gamma _\nu = 1$). In each scenario, the crest height of the embankment is adjusted to reach an overtopping rate of 5 l/m/s under design conditions, which is also used in the CEIP-1 design^83^. The size of the slope protection is calculated using Eq. (11), with *F* = 3.5 (assuming a filter between the clay core and the outer concrete blocks) and *b* = 0.67. The shear stresses at the toe of an embankment are calculated assuming a grain size of $d_{n50} = 7$ $\upmu$m in Eqs. (12–13), corresponding to a muddy coastline. The embankment design parameters are listed in Table 5 of the Supplementary material.

## Results

### Wave energy dissipation as function of forest age

The wave energy dissipation caused by the different parts of a mangrove tree (canopy, trunk, and roots) is shown as a function of tree age in Fig. 5d. For the design water levels at the afforestation sites, the canopy causes most wave energy dissipation during the first 10 years, and has a significant role in wave dissipation until trees are 20 years old. Beyond that age, only trunk and roots contribute to wave dissipation since the canopy emerges from the water (see the submerged areas in Fig. 5c). Trunk and roots still continue to grow for trees older than 30 years old (Fig. 1), and therefore wave dissipation continues to increase over time. Here we have extrapolated trunk growth rates measured in the field for trees with ages between 30 and 50 years old. Assuming no trunk and root growth beyond 30 years of age, the maximum dissipation would be 35 W/m^2^ for a tree at the sea edge of the forest (12% smaller than the maximum value of Fig. 5 d).

The wave height reduction produced by forests of varying widths is shown in Fig. 5e, f. Wave attenuation is here defined as the reduction in wave height at the toe of an embankment (after waves have travelled through the forest) with respect to a situation without mangroves:

16 $$\begin{aligned} \ \frac{\Delta H}{H_{nv}} (\%)= 100 \frac{H_{nv}-H_{v}}{H_{nv}}\ \end{aligned}$$

where $H_{nv}$ is the wave height at the embankment toe without mangrove vegetation (m), and $H_{v}$ is the wave height at the same location considering the presence of a mangrove foreshore (m).

As observed in the wave dissipation rates, wave attenuation varies with tree age. Young trees below 2 years old induce attenuation rates smaller than 30% for all forest widths and wave attenuation increases as trees grow older. For a given age, wider forests increase the wave height reduction rates. For instance, extending the forest width from 100 m to 1000 m increases wave attenuation by a 50-year-old forest from 7% to 40% with $c_{D,w} =$ 0.7 (Fig. 5e). These results are highly sensitive to the drag coefficient. The same forest widths would induce attenuation rates of 20% and 60% with $c_{D,w} =$ 2.0 (Fig. 5f). The large sensitivity to $c_{D,w}$ suggest that good predictions of $c_{D,w}$, of the density $N_v$, and of the tree area are key for precise assessments of the effect of mangroves.

Existing studies investigating the effect of mangroves on coastal protection have focused on the description of the wave climate and used relatively simple descriptions of the geometry. However, the sensitivity analysis (Fig. 6) suggests that the vegetation geometry (Fig. 6a–f) is as important as the hydrodynamic conditions (Fig. 6g–i). For trees younger than 20 years old, since part or all of their canopy is submerged, variations of the canopy height (Fig. 6b), canopy width (Fig. 6e) and tree height (Fig. 6c) induce the largest changes in wave reduction. For shorter species, like those of the Avicenniaceae family, the canopy could have a significant contribution for even longer periods (compared to *S. apetala*). For older *S. apetala* trees, whose canopy is above the water, the trunk diameter has the largest effect on waves (Fig. 6f).

### Vegetation failure

The influence of wind and wave loads on tree stability is illustrated in Fig. 7a. When trees are very young, they remain submerged during storm events and therefore experience wave loads (Fig. 7a). As mangroves grow (see Fig. 5c), they rise above the water surface and the emerged part of the tree is exposed to the wind. For partly submerged mangroves, wind loads are much larger than wave loads (Fig. 7b), since (1) the tree canopy constitutes a large part of the frontal area, and (2) because the (higher) wind speed causes relatively larger forces than the (lower) wave velocities, even if air has a lower density than water. This agrees with field observations by Myers and Lear^58^, who observed that higher inundation levels sheltered trees from wind damage.

*S. apetala* trees younger than 10 years old can thus withstand strong winds without overturning for the embankment design conditions (Fig. 7c), as most of their frontal area is submerged and exposed to the relatively lower wave forces (Fig. 7b). As mangroves grow older they become heavier and more stable but wind forces act over a larger area and with a longer arm. The increase in overturning moment exceeds the increase in tree stability, and taller trees overturn under weaker winds (compared to smaller individuals). Nevertheless, after trees reach their maximum height, trunk and branches continue to grow and mangroves slightly increase their stability over time (Fig. 7c). The resistance against trunk breaking shows a similar behaviour, but the maximum wind speed values are larger (Fig. 7d), indicating that trees would be more likely to topple over than to break for the conditions of Fig. 7c, d.

The maximum wind speeds that mangroves can withstand depend on whether trees can reduce the loads acting on them by bending (Fig. 7c, d). By 30 years old, if trees are fully rigid ($c_{D,c}$ = 1) they can be toppled over by wind speeds between 40-70 km/h, while by being flexible and reducing their frontal area ($c_{D,c}$ = 0.2), they can resist stronger winds up to 100–175 km/h. Drag values oscillated between $c_{D,c}$ = 0.2–1 for western red cedars under winds up to 70 km/h^69^, and they are probably on the lower side of this range (or even below it) for mangroves. Cyclones could induce much larger wind speeds than those tested in wind tunnel experiments^69^, and *S. apetala* trees have a lower mean modulus of elasticity (2500 N/mm^2^)^55^ compared to western red cedars (7095 N/mm^2^)^84^, which suggests that they could be more flexible and may deflect even more under extreme conditions.

### Effect of mangroves on dike design

**Figure 8:**
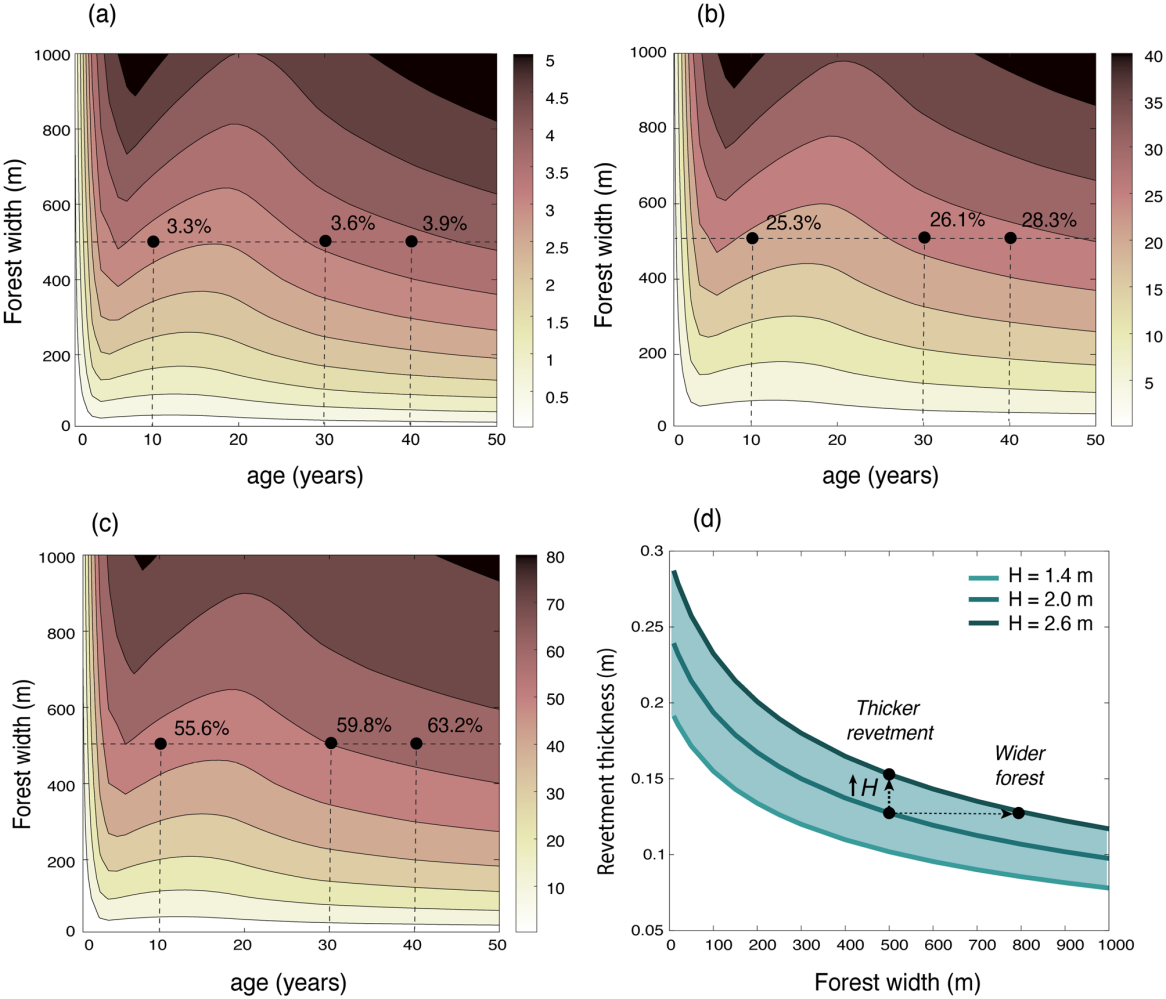
Effect of forest width and age on the reduction of (**a**) embankment crest height, (**b**) thickness of the slope protection, and (**c**) shear stresses at the embankment toe. The bathymetry is assumed linear with a for a bed slope of 1/1000. Vegetation properties in plots (**b**–**d**) correspond with the tree model presented in Fig. 5. In the runs $c_{D,w}$ = 2, $N_v$ = 0.1 trees/m^2^, $H_{m0}$ = 2 m and *h* = 5 m at the embankment toe. (**d**) Sensitivity of the revetment thickness to variations of +/− 20% of the wave height for a tree age of 50-years old.

The embankment height is designed to prevent inundation with total water levels that include tides, surges, and wave-run up. The presence of a mangrove forest has a relatively small effect on embankment crest heights, and, for instance, a 500 m wide forest only reduces the required embankment height by 4% (with an age of 50 years old), while increasing the forest width to 1 km wide only decreases the maximum height by 5% (Fig. 8a). This limited effect is due to surge heights being much larger than the contribution of wave run up, so wave reduction only affects a small part of the design water levels. Nevertheless, even considering surge reduction, narrow mangrove belts also have a very limited effect on surges (with observed reductions of 0–0.25 m per km^10,29–31,45^), implying that narrow mangrove fringes are not efficient in decreasing embankment heights for the cases evaluated in Bangladesh. However, other components of the design are more influenced by wave attenuation—a mangrove belt of 500 m can reduce the revetment thickness by 29% and the bed shear stresses by 65% at 50 years age. An even wider mangrove belt of 1 km would reduce the revetment thickness and shear stresses by 41% and by 83%, respectively. Most of those benefits are already obtained when trees are 10 years old (Fig. 8a–c), due to the fast mangrove growth (Fig. 5c). When the canopy is fully emergent from the water (by approximately 20 years old), the wave attenuation capacity and the effect of mangroves on embankments reaches a relative minimum, but this decrease is compensated by the growth of trunk and roots by the time that trees are 30 years old.

Model results show that the impact of increasing the forest width reduces as the forest becomes wider, since waves attenuate exponentially through the forest^34,85^. However, these results do not include the effect of mangrove failure on wave attenuation predictions.

For a 500-m wide forest, an increase in wave height of 20% due to mangrove damage would imply that the revetment should be 25% thicker or that the forest should be 300 m (60%) wider to compensate for the higher wave exposure (see black arrows in Fig. 8d). It should be noted that fallen trees could still contribute to wave attenuation during the storm event that damaged them, but their effect will disappear over time as they decay.

## Discussion

### Limitations of mangrove geometry models

**Figure 9:**
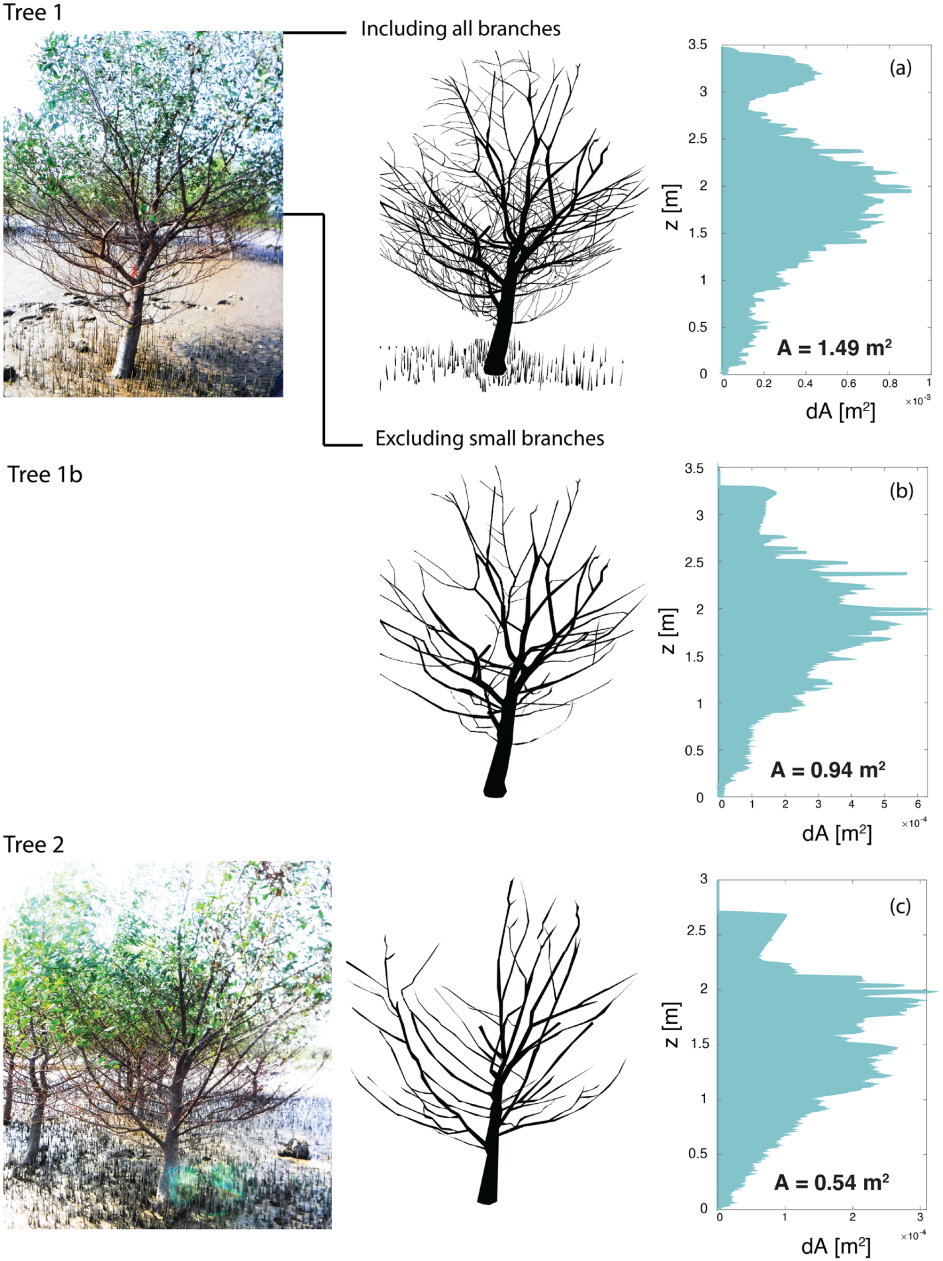
Vertical distribution of the tree area for (**a**) *S. apetala* tree area of the top left picture (tree 1), of (**b**) the same tree excluding the smaller branches (tree 1b), and for (**c**) another *S. apetala* tree from a picture with higher contrast (tree 2), modified from Gijón Mancheño et al.^50^.

The tree geometry model presented in this study (Fig. 5b) is based on two young *S. apetala* specimens (Fig. 9), and additional data collection is recommended to include more samples and cover a wider range of ages. The mangroves evaluated in this study are probably around 3 years old (based on the height-age relationship from Figure 1), and their stem region is therefore narrow, with the canopy extending over most of their height. A similar vertical distribution was observed for the solid volume of 4-m tall Chinese *S. apetala* trees with estimated ages between 2 and 6 years old (see Figure 1 e of Zhang et al.^78^), which resembled bushes more than trees. However, older trees *S. apetala* from Google images^86,87^ display a more distinct stem layer below the canopy, and the vertical distribution of their surface area could differ from Fig. 5b. Measuring the tree surface area for different ages would enable assessing how the canopy changes as *S. apetala* trees grow, and how it varies between different individuals.

The natural variability in surface area for *S. apetala* trees of the same age could not be evaluated in this work, but their variability in height has been reported in the literature. In the Sundarbans, Rahman et al.^25^ found that *S. apetala* trees had mean heights of 17.97 m with a standard deviation of +/− 5.9 m (corresponding with deviations of +/− 30% in height). According to our sensitivity analysis, +/− 30% in tree height (compared to our reference tree model, shown in Fig. 5b) could cause up to 16% differences in wave height reduction for 500-m belts more than 30 years old (Fig. 6c), and much larger differences for younger fringes. A 30% deviation in the diameter at breast height would cause an even larger effect, up to 29% change in wave reduction (Fig. 6f). The changes in different tree properties could be correlated (for instance, shorter trees could spend their resources into building thicker trunks and denser canopies), and some effects could cancel each other out or add up. Understanding such dependencies would enable better predictions of the performance of a natural forest.

The net effect of natural variability can differ considerably between locations, as it will depend on the causes of the differences among individuals, the relative location of the trees, and the relative water level at such locations. For instance, thicker and denser trees could increase the submerged biomass and induce larger wave attenuation^34^. However, their effect on wave reduction by a forest would be larger if they are present closer to the sea side, where most short wave attenuation takes place^85^. Since there are numerous factors simultaneously affecting vegetation growth, the cumulative effect of the variations in vegetation geometry is likely site-dependent.

In this study, we explore the effect of a mangrove belt formed by *S. apetala* trees of equal age, but geometry and stability models for more species would be needed to simulate a biodiverse forest. Geometry and growth models could also be expanded to reproduce differences in growth for a single species as a function of site-dependent environmental conditions^45^, and to improve the description of individual trees (e.g., changing the vertical distribution of the surface area with tree age, or including the effect of processes like competition and self-thinning^88^). With such models, given some general information about the forest population (such as distribution of species and tree ages), it would be possible to calculate the proportion of trees that could be damaged during a storm, and to estimate how wide a forest should be to compensate for tree losses.

### Limitations of mangrove failure model

Model results suggest that, both with or without flexibility effects, *S. apetala* trees could overturn for wind speeds typical of cyclones in Bangladesh^16,17,59,61,62^. The threshold wind speeds for trunk breaking are higher than for overturning, indicating that trees are more likely to topple over than to experience stem breaking. However, such results are obtained using values for overturning resistance for terrestrial trees^72^. On the one hand, mangroves are often found at muddy sites with soft sediment that could lead to lower $C_r$ values, but on the other hand, they have complex root systems that could provide extra stability compared to terrestrial species. Erosive and accretive processes will also affect the chances of failure, since they can modify the resisting moment of the tree. Moreover, we schematize the overturning strength as a function of the tree weight^72^, but the shape and size of the root system will also influence tree stability^65^. Post-cyclone field observations and tree pulling experiments would be very valuable to expand the failure mechanisms and develop precise models of mangrove stability.

Forces are also modelled in a simplified manner that could be developed in follow-up studies. For instance, dynamic effects^89^ are neglected and we only model forces experienced at the edge of a forest (without considering sheltering effects between trees^64^). Wind tunnel experiments with mangrove trees are also advised to study their motion under strong winds (e.g., as done by Rudnicki et al.^69^ with terrestrial trees), and to define the range of drag values which can be expected. Not all components of the tree will contribute equally to wind and wave loads, as more flexible elements can reduce the forces acting on them by swaying and bending^54,90^. However, the degree of force reduction of different mangrove branches as a function of their size and flexibility^65^ has not yet been investigated.

Both model results and field observations^16,17,59,61,62^ suggest that mangroves can be harmed during cyclones, and different strategies can be considered to mitigate damages. Smaller trees have been found to be more resistant against failure due to their lower exposure to wind^59^, and the relatively shorter pioneer species *Avicennia officinalis* may thus have an advantage during extreme conditions. At managed forests, crown tapering^91^ above the design water levels may be a method to prevent mangrove losses.

### Surge reduction by mangroves

The effect of mangroves on embankment heights is only evaluated for wave processes, whereas we have neglected surge reduction by mangroves. In our scenarios, for the most favorable case of a 1-km mangrove belt with uniform vegetation and no channels, extrapolating the largest surge reduction observed in the field^31^, the maximum surge height reduction would be 0.25 m. Since the design water levels at the selected sites in Bangladesh reach 5 m, the maximum water level reduction would be 5%. Narrower mangrove belts would have even less effect on the water levels^30,31^, as supported by the modelling study of Dasgupta et al.^10^, who obtained negligible surge reduction by 500-m wide mangrove belts in Bangladesh. At sites with multiple tidal channels, the surge reduction could be negligible for all forest widths, as observed by Montgomery et al.^27^ in a mangrove forest at Tauranga harbour (New Zealand).

Wider forests, of several kilometers, could induce a larger reduction of the design water levels^27,31^ and the percentage of surge reduction could also be larger for lower water levels^29,30^. At locations with relatively lower surges, wave run up could have a larger relative contribution on the design levels, and mangrove belts could be more efficient in lowering embankment heights. Surge reduction by mangroves is a highly site-specific process^27–31^, very dependent on the local topography and vegetation properties, and quantifying this process thus requires applying flow models, such as Delft3D^92^, MIKE 21^10^ or SFINCS^93^, in combination with local topographic and vegetation data. Such models could also estimate current reduction by mangroves, which was found to be significant even for relatively narrow mangrove fringes in Bangladesh by Dasgupta et al.^10^.

### Implementation of mangroves into embankment designs

The methodology presented in this study provides a foundation to assess the potential effect of a mangrove belt on embankment designs, and to evaluate whether mangroves would be stable during design conditions. At a given site, the steps would be the following: (1) developing tree models (e.g., as those shown in Fig. 5) of the local pioneer species and defining the mangrove scenarios (species, density, and location along the profile as a function of time), (2) defining the design conditions of the coastal embankments (local bathymetry, water levels, wind and wave climate, as listed in Table 1 and Table 5 of the Supplementary material), (3) assessing the stability of mangroves during the design conditions (Fig. 7), (4) estimating wave reduction by a potential mangrove belt and its effect on the embankment design (as in Figs. 5e, f and 8).

As previously discussed, if the wind speeds during design conditions exceed the vegetation resistance, different combinations of the local pioneers (giving preference to relatively shorter species) and/or tapering measures could be evaluated, and steps (1)–(4) could be repeated for such scenarios. If the vegetation is not stable for any possible configuration, flood protection structures should be designed to prevent flooding on their own. It could then be investigated if the vegetation could still be implemented to provide additional safety (e.g., by enhancing sedimentation during calmer periods), considering its degradation during extreme events and its posterior regeneration. At sites that are initially unvegetated, embankments could be designed to fully protect from flooding at the beginning of the structure lifetime, while wide mangrove belts could be implemented to reduce the increase in water levels due to climate change, or to decrease the frequency of maintenance works.

Once a target forest width is defined (for natural colonization, restoration or afforestation), the forest should be monitored and managed over the lifetime of the design, conducting preventive measures before storm or cyclone seasons or temporarily reinforcing the embankments while the mangrove forest is recovering from storm damage. Potential damage by pests would also imply that forests may need to be even wider and more biodiverse (containing several species) to provide sufficient safety. An additional buffer may also be needed to account for habitat squeezing due to sea level rise. Overall, the choice of the optimal mangrove width is site-specific and requires balancing additional factors such as other ecosystem services, and the costs and benefits of other land use alternatives.

## Conclusions

This paper presents a model to evaluate the effect of wave attenuation by mangroves on embankment designs, including tree growth and failure due to winds and waves. The model is applied to potential afforestation sites seaward of coastal embankments in Bangladesh, using the properties of the local mangrove pioneer *Sonneratia apetala*. Model results indicate that wave attenuation predictions are very dependent on the tree surface area. Accurate descriptions of the canopy are necessary for *Sonneratia apetala* trees younger than 20 years old for design water levels of 5 m, whereas the trunk diameter is most important for older trees. Mangrove fringes with cross-shore widths < 1 km have most effect on the slope protection and on the bed erodibility at the embankment toe. However, the effect of wave reduction by such mangrove belts on embankment crest heights is small, since embankment overtopping is dominated by the large surge levels at the potential afforestation sites in Bangladesh. Comparison of overturning moments by winds with the resisting moments of mangroves suggest s that *S. apetala* trees could collapse for wind speeds between 85 and 260 km/h, which are frequently caused by cyclones in Bangladesh. Implementing biodiverse forests with shorter pioneers like *Avicennia officinalis* or tree tapering before cyclone seasons could reduce the chance of tree failure.

## Methods

### Quantifying mangrove geometry

Predicting wave attenuation by mangroves requires describing their frontal surface area, including roots, trunk, and canopy. A literature study (Fig. 1 and Table 1) provides information about the trunk and roots of *S. apetala* mangroves, but no information was found for the canopy. The canopy area is thus obtained by digitizing images of *S. apetala* trees collected in China. The images are scaled assuming a pneumatophore height of 15 cm, which was the average value observed in the area. Overexposure and shadowing resulted in branches and leaves being partly colored and partly black and white, which hinders separating the leave and branch area using color thresholds. Therefore, the contour of the trunk and branches is manually digitized in Photoshop and read as a binary image in MATLAB. Over the height of the tree, the number of pixels corresponding with the tree area are summed to obtain the vertical distribution of the exposed surface. The total tree area is divided by the tree height to calculate an equivalent tree width $b_v$, which is normalized using the $d_{BH}$ of the tree $(b_v/d_{BH})$. Since branches with diameters smaller than 1 cm cannot always be identified in the pictures due to inhomogenous lighting, the image analysis focuses on the image with best resolution and colour homogeneity (Fig. 9, 1), and the results are compared with a second specimen photographed with less quality (Fig. 9, 2). The resulting tree properties are summarized in Table 2.

**Table 2:** Tree properties of *Sonneratia apetala* specimens from field pictures, consisting of the tree frontal area *A*, the diameter at breast height $d_{BH}$, the vegetation height $h_v$, the elevation at which the canopy starts compared to the tree height $h_c/h_v$, and the equivalent width of the tree, $b_{v} = A/(d_{BH} h_v)$, compared to the diameter at breast height.

Specimen	*A* [m^2^]	$$d_{BH}$$ [m]	$$h_v$$ [m]	$$h_c/h_v$$ [-]	$$b_{v}/d_{BH} = A/(h_v d_{BH}) $$ [m] [-]
Tree 1	1.50	0.13	3.46	0.29	3.0
Tree 1b	0.94	0.13	3.46	0.29	2.1
Tree 2	0.56	0.08	2.62	0.12	2.6

For the highest quality picture the total tree area (neglecting leaves) is 1.5 m^2^ (Fig. 9a), with a diameter at breast height of $d_{BH}$ = 0.13 m and a tree height of $h_v$ = 3.46 m. The canopy starts at $h_{c}$ = 1 m from the ground, corresponding with 29% of the tree height ($h_{c}$ = 0.29 $h_v$), although this value could be underpredicted due to the perspective of the picture. The canopy has a total area of 1.35 m^2^ including the trunk, and 1.19 m^2^ excluding it. Therefore 90% of the tree frontal area is in the canopy, and 80% in the canopy branches.

The size and location of the pneumatophores is distorted, causing an underestimation of the root area at every height. In view of Table 1, assuming a root density of $N_r$ = 100–200 roots/m^2^, a root height of $h_r =$ 0.1–0.2 m, a root diameter of $d_r =$ 7 mm, and that the roots extend over a radius of 2 m from the trunk, the total frontal area of the roots would range between $A_c$ = 0.2-0.9 $m^2$, which would increase the tree frontal area in 30-60% with respect to the estimates of Table 2. Removing the the branches smaller than 1 cm and the pneumatophores (Fig. 9b) reduces the total tree area from $A=$ 1.5 m^2^ to A = 0.94 m^2^ (in 37%).

A second *S. apetala* specimen where the smallest branches are not distinguisible due to lower image quality (Fig. 9, 2) has a total area of $$A = $$ 0.56 m^2^ (Fig. 9c), a diameter at breast height of $$d_{BH}$$ = 0.08 m, and a tree height of $$h_v$$ = 2.62 m. For the second specimen, the canopy starts at $$h_{c} = $$ 0.32 m above the ground, corresponding with 12% of the tree height ($$h_{c} = 0.12 h_v$$). The vertical distribution of the tree areas shows a maximum at the canopy, even neglecting the leaves and the smallest branches. In this analysis, small branches are neglected and leaves are also disregarded under the assumption that they are quite flexible and easily detachable^55^. Despite their flexibility, these elements likely contribute to some extent to wave dissipation by the trees.

### Supplementary Information

Supplementary Information.

## Data Availability

The datasets used and/or analysed during the current study available from the corresponding author on reasonable request.

## References

[1] McIvor, A., Möller, I., Spencer, T. & Spalding, M. Reduction of wind and swell waves by mangroves. Natural coastal protection series: Report 1. Tech. Rep., Cambridge coastal research unit working paper 40. In *The Nature Conservancy, Arlington, USA/Wetlands International, Wageningen, Netherlands* 27 (2012).

[2] Menéndez P, Losada I, Torres-Ortega S, Narayan S, Beck M (2020). The global flood protection benefits of mangroves. Sci. Rep..

[3] Van Zelst V, Dijkstra J, Van Wesenbeeck B (2021). Cutting the costs of coastal protection by integrating vegetation in flood defences. Nat. Commun..

[4] Tiggeloven T (2022). The benefits of coastal adaptation through conservation of foreshore vegetation. J. Flood Risk Manage..

[5] Brander L (2012). Ecosystem service values for mangroves in Southeast Asia: A meta-analysis and value transfer application. Ecosyst. Serv..

[6] Woodroffe C (2016). Mangrove sedimentation and response to relative sea-level rise. Annu. Rev. Mar. Sci..

[7] Lovelock C (2015). The vulnerability of Indo-Pacific mangrove forests to sea-level rise. Nature.

[8] Saintilan N (2020). Thresholds of mangrove survival under rapid sea level rise. Science.

[9] Murdiyarso D (2015). The potential of Indonesian mangrove forests for global climate change mitigation. Nat. Clim. Chang..

[10] Dasgupta, S. *et al.* Vulnerability of bangladesh to cyclones in a changing climate: Potential damages and adaptation costs. In Tech. Rep., Policy Research Working aper 5280, The World Bank Development Research Group, Environment and Energy Team (2010).

[11] Srikanth S, Lum S, Chen Z (2016). Mangrove root: Adaptations and ecological importance. Trees.

[12] Snedaker S (1982). Mangrove Species Zonation: Why? Contributions to the Ecology of Halophytes.

[13] Ellison A, Mukherjee B, Karim A (2000). Testing patterns of zonation in mangroves: Scale dependence and environmental correlates in the Sundarbans of Bangladesh. J. Ecol..

[14] Bunt J (1996). Mangrove zonation: An examination of data from seventeen riverine estuaries in tropical Australia. Ann. Bot..

[15] Lopez-Portillo J, Ezcurra E (1981). Zonation in mangrove and salt marsh vegetation at laguna de Mecoacan, Mexico. Biotropica.

[16] Carrol C, Bunting P, Hardy A, Bell G (2019). Using continuous change detection and classification of landsat data to investigate long-term mangrove dynamics in the sundarbans region. Remote Sens..

[17] Bhowmik A, Cabral P (2013). Cyclone sidr impacts on the sundarbans floristic diversity. Earth Sci. Res..

[18] Eckstein D, Künzel V, Schäfer L, Winges M (2020). Global climate risk index 2019. Germanwatch e.V..

[19] Saenger P, Siddiqi N (1993). Land from the sea: The mangrove afforestation program of Bangladesh. Ocean Coast. Manag..

[20] Gijón Mancheño A (2021). Mapping mangrove opportunities with open access data: A case study for Bangladesh. Sustainability.

[21] Jakobsen F, Zeaul-Hoque A, Paudyal G, Bhuiyan MS (2017). Bringing in the tides. From closing down to opening up delta polders via Tidal River Management in the southwest delta of Bangladesh. Water Policy.

[22] Vuik V, Jonkman S, Borsje B, Suzuki T (2016). Nature-based flood protection: The efficiency of vegetated foreshores for reducing wave loads on coastal dikes. Coast. Eng..

[23] Vuik V (2018). Assessing safety of nature-based flood defenses: Dealing with extremes and uncertainties. Coast. Eng..

[24] Vuik V, Borsje B, Willemsen P, Jonkman S (2019). Salt marshes for flood risk reduction: Quantifying long-term effectiveness and life-cycle costs. Ocean Coast. Manag..

[25] Rahman M, Zimmer M, Ahmed I (2021). Co-benefits of protecting mangroves for biodiversity conservation and carbon storage. Nat. Commun..

[26] Wang Q (2019). Sequestration of heavy metal by glomalin-related soil protein: Implication for water quality improvement in mangrove wetlands. Water Res..

[27] Montgomery J, Bryan K, Horstman E, Mullarney J (2018). Attenuation of tides and surges by mangroves: Contrasting case studies from New Zealand. Water.

[28] Krauss K (2009). Water level observations in mangrove swamps during two hurricanes in Florida. Wetlands.

[29] Gedan K, Kirwan M, Wolanski E, Barbier E, Silliman B (2011). The present and future role of coastal wetland vegetation in protecting shorelines: Answering recent challenges to the paradigm. Clim. Change.

[30] Temmerman S (2023). Marshes and mangroves as nature-based coastal storm buffers. Annu. Rev. Mar. Sci..

[31] McGee, B., Goree, B., Tollett, R., Woodward, B. & Kress, W. Hurricane rita surge data, southwestern louisiana and southeastern texas, 2005. In *US Geological Survey Data Series 220* (2006).

[32] Mazda Y, Wolanski E, King B (1997). Drag force due to vegetation in mangrove swamps. Mangrove Salt Marshes.

[33] Quartel S, Kroon A, Augustinus P, Van Santen P, Tri N (2007). Wave attenuation in coastal mangroves in the red river delta, Vietnam. J. Asian Earth Sci..

[34] Bao T (2011). Effect of mangrove forest structures on wave attenuation in coastal Vietnam. Oceanologia.

[35] Horstman E (2014). Wave attenuation in mangroves: A quantitative approach to field observations. Coast. Eng..

[36] Méndez F, Losada I (2004). An empirical model to estimate the propagation of random breaking and nonbreaking waves over vegetation fields. Coast. Eng..

[37] Uddin M, Hossain M (2013). Growth performance of coastal plantations and land stabilization in an offshore island of Hatiya, Noakhali, Bangladesh. Bangladesh J. Forest Sci..

[38] Islam S, Miah S, Habib M, Alam M (2016). Growth and yield of sonneratia apetala (keora) plantations raised from different seed sources in the central coastal belt of Bangladesh. J. Biosci. Agric. Res..

[39] Van der Meer, J. *et al.* Manual on wave overtopping of sea defences and related structures. Tech. Rep., An overtopping manual largely based on European resreach, but for worldwide application. www.overtopping-manual.com: EurOtop (2018).

[40] Pilarczyk K (1990). Coastal Protection.

[41] Pilarczyk K (1998). Dikes and Revetments.

[42] Wang W, Li X, Wang M (2019). Propagule dispersal determines mangrove zonation at intertidal and estuarine scales. Forests.

[43] Balke T (2011). Windows of opportunity: Thresholds to mangrove seedling establishment on tidal flats. Mar. Ecol. Prog. Ser..

[44] Maza M, Lara J, Losada I (2021). Predicting the evolution of coastal protection service with mangrove forest age. Coast. Eng..

[45] Krauss K (2008). Environmental drivers in mangrove establishment and early development: A review. Aquat. Bot..

[46] Rahman M, Sass-Klaassen U, Zuidema P, Chowdhury M, Beeckman H (2020). Salinity drives growth dynamics of the mangrove tree sonneratia apetala in the sundarbans, bangladesh. Dendrochronologia.

[47] Simard M, Fatoyinbo L, Smetanka CEA (2019). Mangrove canopy height globally related to precipitation, temperature and cyclone frequency. Nat. Geosci..

[48] Wang G, Zhang Y, Guan D, Xiao L, Singh M (2021). The potential of mature sonneratia apetala plantations to enhance carbon stocks in the zhanjiang mangrove national nature reserve. Ecol. Ind..

[49] Xin K, Zhou Q, Arndt S, Yang X (2013). Invasive capacity of the manrgove sonneratia apetala in hainan island, china. J. Trop. For. Sci..

[50] Gijón Mancheño, A. *et al.* Wave reduction by mangroves during cyclones in bangladesh. Tech. Rep., Policy Research Working Paper 10240 (2021).

[51] Zhang Y (2021). Non-linear wave attenuation quantification model improves the estimation of wave attenuation efficiency of mangroves. Estuar. Coast. Shelf Sci..

[52] Suzuki T, Zijlema M, Burger B, Meijer M, Narayan S (2012). Wave dissipation by vegetation with layer schematization in SWAN. Coast. Eng..

[53] Massel S, Furukawa K, Brinkman R (1999). Surface wave propagation in mangrove forests. Fluid Dyn. Res..

[54] Van Wesenbeeck B (2022). Wave attenuation through forests under extreme conditions. Sci. Rep..

[55] Van Hespen R (2021). Analysis of coastal storm damage resistance in successional mangrove species. Limnol. Oceanogr..

[56] Kalloe S, Hofland B, Antolínez J, van Wesenbeeck B (2022). Quantifying frontal- surface area of woody vegetation: A crucial parameter for wave attenuation. Front. Mar. Sci..

[57] Lopez-Portillo, J., Lewis III, R., Saenger, P. & Rovai, A. *Mangrove Forest Restoration and Rehabilitation. Mangrove Ecosystems: A Global Biogeographic Perspective* (Springer, 2017).

[58] Myers R, van Lear D (1998). Hurricane-fire interactions in coastal forests of the south: A review and hypothesis. For. Ecol. Manage..

[59] Krauss K, Osland M (2020). Tropical cyclones and the organization of mangrove forests: A review. Ann. Bot..

[60] Herrera-Silveira, J. A. *et al.**Hurricane Damages to Mangrove Forests and Post-Storm Restoration Techniques and Costs* (The Nature Conservancy, 2022).

[61] Mandal M, Hosaka T (2020). Assessing cyclone disturbances (1988–2016) in the sundarbans mangrove forests using landsat and google earth engine. Nat. Hazards.

[62] Dutta D, Das P, Paul S, Sharma R, Dadhwal V (2015). Hassessment of ecological disturbance in the mangrove forest of sundarbans caused by cyclones using modis time-series data (2001–2011). Nat. Hazards.

[63] Pendleton L (2012). Estimating global blue carbon emissions from conversion and degradation of vegetated coastal ecosystems. PLoS ONE.

[64] Gardiner B, Berry P, Moulia B (2016). Review: Wind impacts on plant growth, mechanics and damage. Plant Sci..

[65] van Hespen, R. *Establishment and Survival of Coastal Mangrove Trees Under Mechanical Disturbances*. Ph.D. thesis, University of Utrecht (2023).

[66] Mo Y, Simard M, Hall J (2023). Tropical cyclone risk to global mangrove ecosystems: Potential future regional shifts. Front. Ecol. Environ..

[67] Thornton E, Guza R (1983). Transformation of wave height distribution. J. Geophys. Res..

[68] Morison JR, O’Brien MP, Johnson JW, Schaaf SA (1950). The force exerted by surface waves on piles. Petroleum Trans..

[69] Rudnicki M, Mitchell S, Novak M (2004). Wind tunnel measurements of crown streamlining and drag relationships for three conifer species. Can. J. For. Res..

[70] Bardal L, Saetran L (2016). Wind gust factors in a coastal wind climate. Energy Procedia.

[71] Quine P, Gardiner B (2007). Understanding how the interaction of wind and trees results in windthrow, stem breakage and canopy gap formation draft of 2/08/05. Plant Disturb. Ecol..

[72] Nicoll B, Gardiner B, Rayner B, Peace A (2016). Anchorage of coniferous trees in relation to species soil type, and rooting depth. Can. J. For. Res..

[73] Khan Z (2016). Disaster impact on sundarbans: A case study on sidr affected area. Int. J. Res. Appl. Nat. Soc. Sci..

[74] Ghosh M, Kumar L, Langat P (2019). Geospatial modelling of the inundation levels in the sundarbans mangrove forests due to the impact of sea level rise and identification of affected species and regions. Geomat. Nat. Haz. Risk.

[75] Tanaka, N. Investigation team of japan society of civil engineering. In *I*nvestigation Report on the Stormsurge Disaster by Cyclone Sidr in 2007, Bangladesh. Tech. Rep., JSCE (2011).

[76] Hussain M, Tajima Y, Hossain M, Das P (2017). Impact of cyclone track features and tidal phase shift upon surge characteristics in the Bay of Bengal along the Bangladesh coast. J. Mar. Sci. Eng..

[77] Haque A, Kay S, Nicholls R (2018). Present and future fluvial, tidal and storm surge flooding in coastal Bangladesh. Ecosyst. Serv. Well-Being Deltas.

[78] Zhang X, Lin P, Chen X (2022). Coastal protection by planted mangrove forest during typhoon mangkhut. J. Mar. Sci. Eng..

[79] Zhu D, Hui D, Wang MEA (2021). Allometric growth and carbon storage in the mangrove sonneratia apetala. Wetlands Ecol. Manage..

[80] Peterson T, Cannon J (2021). Modelling wind damage to southeastern us trees: Effects of wind profile, gaps, neighborhood interactions, and wind direction. Front. For. Glob. Change.

[81] D-Morphology User Manual. *Version 2023 of 8th December 2022* (2022).

[82] IWM. Technical report on storm surge, wave, hydrodynamic modelling and design parameters on drainage system and embankment crest level. volume-ii: Package 2 (appendix-b: Storm surge). Tech. Rep., Bangladesh Water Development Board, Ministry of Water Resources (2018).

[83] Of Bangladesh, G. Detailed design of five polders. volume i: Design report. Tech. Rep., Government of Bangladesh (2012).

[84] Molinkski W, Mania P, Tomczuk G (2016). The usefulness of different wood species for bow manufacturing. Folia Forestalia Polon..

[85] Dalrymple R, Kirby J, Hwang P (1984). Wave diffraction due to areas of energy dissipation. J. Waterw. Port Coast. Ocean Eng..

[86] Picture of Sonneratia apetala mangroves in the Sundarbans. http://sundarban007.blogspot.com/2013/05/sonneratia-apetalakeora.html (2022).

[87] Picture of Sonneratia apetala plantation. https://www.cabidigitallibrary.org/doi/full/10.1079/cabicompendium.50590 (2023).

[88] Chen L (2021). Forest thinning in the seaward fringe speeds up surface elevation increment and carbon accumulation in managed mangrove forests. J. Appl. Ecol..

[89] James K, Haritos N, Ades P (2006). Mechanical stability of trees under dynamic loads. Physiol. Biochem..

[90] Luhar M, Nepf H (2011). Flow-induced reconfiguration of buoyant and flexible aquatic vegetation. Limnol. Oceonogr..

[91] Halder N, Merchant A, Misbahuzzaman K, Wagner S, Mukul S (2021). Why some trees are more vulnerable during catastrophic cyclone events in the sundarbans mangrove forest of bangladesh?. For. Ecol. Manage..

[92] Hu K, Chen Q, Wang H (2015). A numerical study of vegetation impact on reducing storm surge by wetlands in a semi-enclosed estuary. Coast. Eng..

[93] Leijnse T, van Ormondt M, Nederhoff K, van Dongeren A (2021). Modeling compound flooding in coastal systems using a computationally efficient reduced-physics solver: Including fluvial, pluvial, tidal, wind- and wave-driven processes. Coast. Eng..

