# Supplementary material for: Integrating mangrove growth and failure in coastal flood protection designs
Source: Sci Rep. 2024 Apr 4;14:7951. doi: 10.1038/s41598-024-58705-4 (PMC10995189; doi:10.1038/s41598-024-58705-4)
Supplement: Supplementary file 1 — Supplementary Information. [file 41598_2024_58705_MOESM1_ESM.pdf]

Supplementary material

Integrating mangrove growth and failure in flood protection designs

Gijón Mancheño, A.[a], Vuik, V.[a,b], van Wesenbeeck, B.K.[a, c], Jonkman, S.N.[a], van Hespen, R. [d], Moll, J.R.[a], Kazi, S.[e], Urrutia, I.[e], van Ledden, M[e].

[a] *Delft University of Technology, Stevinweg 1, Delft, 2628CN, the Netherlands*

[b] *HKV Consultants, P.O. Box 2120, Lelystad, 8203AC, the Netherlands*

[c] *Department of Ecosystems and Sediment Dynamics, Deltares, P.O. Box 177, Delft, 2600MH, the Netherlands*

[d] *Department of Estuarine and Delta Systems, WNIOZ Yerseke, Royal Netherlands Institute for Sea Research and Utrecht University, Netherlands*

[e] *World Bank, 1818 H Street, 20433, Washington DC, USA*

This supplementary material section provides additional information about the meaning and values of the parameters included in the wave model, wind model, mangrove tree surface area model, mangrove tree failure model, and embankment design model. The values of the parameters correspond with those used in the Results sections. We have included the range of values of the inputs and of the intermediate variables used in the formulas.

Table S1. Meaning and values of the parameters of the wave attenuation and wave load model

Model	Symbol	Name	Unit	Meaning	Value	Equation	Type
Wave model	x	Cross-shore coordinate	*m*	Cross-shore coordinate perpendicular to the coastline	0-1000	1-4	Variable
	z	Vertical coordinate	*m*	Vertical coordinate (from sea surface to sea bottom)	0-5	1-4	Variable
	t	Time	*s*	Time coordinate	0-5,2	1-4	Variable
	h	Water depth	*m*	Distance between water surface and sea bottom	3,5-6,5	1-4	Input
	θ	Wave angle	*radians*	Angle of wave attack - waves parallel to the shoreline have θ = 0	1,6	1	Input
	Hrms	Wave height	*m*	Root-mean-square wave height at offshore boundary (from wave spectrum)	1,4-2,6	1-2	Input
	fp	Wave peak frequency	*s-1*	Peak wave frequency (from wave spectrum)	0,19-0,24	1-3	Input
	Tp	Wave peak period	*s*	Peak wave period (from wave spectrum), equal to 1/fp	4,3-5,2	1-4	Input
	E	Wave Energy	*J/m2*	Wave energy of individual wave fronts per unit area	1168-5518	1	Output
	cg	Group celerity	*m/s*	Propagation speed of a group of waves	4-5	1	Output
	εb	Dissipation by breaking	*W/m2*	Wave energy loss due to wave breaking	0-80	1-2	Output
	ρw	Water density	*kg/m3*	Density of sea water	1030	3-4	Input
	g	Gravity	*m/s2*	Acceleration of gravity	9,81	2	Input
	B	Breaker coefficient	-	Empirical parameter	1	2	Input
	γbr	Breaker coefficient	-	Empirical parameter	0,6	2	Input
	εv	Dissipation by vegetation	*W/m2*	Wave energy loss due to the presence of aquatic vegetation	2-24	4	Output
	Fw	Wave force on vegetation	*N*	Force exerted by waves on submerged vegetation	-	3	Output
	cDw	Drag coefficient (waves)	-	Drag coefficient of mangrove branches under wave loads	0,7-2	3	Input
	cM	Inertia coefficient	-	Inertia coefficient of mangrove branches under wave loads	2	3	Input
	uw	Wave orbital velocity	*m/s*	Cross-shore wave-driven orbital velocity	0,4-1,8	3	Input
	duw/dt	Wave orbital acceleration	*m/s2*	Cross-shore wave driven orbital acceleration	0,5-2,2	3	Input
	bv	Equivalent width	*m*	Cummulative width of all branches or roots at a height z from the ground	0-2,1	3-4	Input
	hv	Tree height	*m*	Height of a tree, from the ground to the highest branch	0-18	3-4	Input
	Nv	Tree density	*m-2*	Number of trees per unit area	0,1	4	Input

Table S2. Meaning and values of the parameters of the wind load model

Model	Symbol	Name	Unit	Meaning	Value	Equation	Type
Wind model	z	Vertical coordinate	*m*	Vertical coordinate (from sea surface to sea bottom)	0-18	5-6	Variable
	Fa	Wave force on vegetation	*N*	Force exerted by wind on emergent vegetation	0-13036	5	Output
	cDc	Drag coefficient (wind)	-	Drag coefficient of mangrove branches under wind loads	0,2-1	5	Input
	G	Gust factor	-	Ratio between maximum gust speed and mean wind speed	1,2	5	Input
	ua	Wind speed	*m/s*	Horizontal wind speed 10 m from the ground, over a period of 1-3 minutes	27-72	5	Input
	ua*	Friction velocity	*m/s*	Expression of wind shear stress as a velocity	4-24	6	Input
	k	Von Karman constant	-	Constant in the logarithmic velocity profile	0,4	6	Input
	z0	Roughness height	*m*	Ground roughness height, equal to 0,002 m for water	0,002	6	Input
	bv	Branch width	*m*	Cummulative width of all branches in a vertical layer	0-2	5	Input
	hv	Tree height	*m*	Height of a tree, from the ground to the highest branch	0-18	5	Input
	ρa	Air density	*kg/m3*	Density of air	1,2	5	Input

Table S3. Meaning and values of the mangrove tree surface area model

Model	Symbol	Name	Unit	Meaning	Value	Equation	Type
Tree model	z	Vertical coordinate	*m*	Vertical coordinate (from sea surface to sea bottom)	0-18	3-5	Variable
	bv	Branch width	*m*	Sum of all branch widths at a given height from the ground	0-3,6	3-5	Input
	dBH	Diameter at breast height	*m*	Trunk diameter at breast height (1/3 from the ground)	0-0,3	3-5	Input
	hv	Tree height	*m*	Height of a tree, from the ground to the highest branch	0-18	3-5	Input
	hr	Root height	*m*	Height of the pneumatophore layer	0-0,7	3-5	Input
	dr	Root diameter	*m*	Mean diameter of pneumatophores	0-3,6	3-5	Input
	hc	Canopy height	*m*	Height at which canopy starts	0-6	3-5	Input
	dc	Canopy width	*m*	Maximum width of the canopy	0-2,1	3-5	Input

Table S4. Meaning and values of the mangrove tree failure model

Model	Symbol	Name	Unit	Meaning	Value	Equation	Type
Failure model	M	Overturning moment	*Nm*	Total overturning moment by wind and waves on a tree	8-151100	7	Output
	Mbreak	Resisting moment to breaking	*Nm*	Maximum overturning moment that a tree can withstand before toppling over	84-100020	7,8	Output
	Mover	Resisting moment to overtoppling	*Nm*	Maximum overturning moment that a tree can withstand before breaking	126-6269	7,9	Output
	F	Total force on tree	*N*	Total force on a tree (sum of wind and wave loads)	7-13171	7	Output
	σu	Modulus of rupture wood tissue	*N/mm2*	Maximum shear stress that a branch can resist without breaking	37 +/- 7	8	Input
	fknot	Strength reduction due to knots	-	Reduction factor for σu, to account for the presence of knots	1	8	Input
	dBH	Diameter at breast height	*m*	Trunk diameter at one third of the tree height	0-0,3	8	Input
	Cr	Regression constant	*m2/s2*	Regression constant for the resistance against overtoppling	60-200	9	Input
	W	Tree weight	*kg*	Weight of a mangrove tree	2-313	9	Input

Table S5. Meaning and values of the embankment design model

Model	Symbol	Name	Unit	Meaning	Value	Equation	Type
Embankment design model	q	Overtoppling discharge per meter	*m3/m/s*	Overtopping discharge (in every scenario hcrest is chosen so that q = 5 l/m/s)	0,005	10	Output
	g	Gravity	*m/s2*	Acceleration of gravity	9,81	10	Input
	Hm0	Wave height	*m*	Significant wave height with wave reduction by forest (Hm0 = Hrms/0,7)	1,03-1,21	10	Input
	f1	Empirical factor	-	Empirical factor in the overtopping discharge formulation	0,026	10	Input
	α	Angle of outer slope	*rad*	Angle of the embankment slope with respect to the ground	0,12	10	Input
	γb	Influence factor for the berm	-	Influence factor for the berm (1 if no berm)	0,89	10	Input
	εm-1.0	Breaker parameter	-	Breaker parameter, representing the type of wave breaking on the slope	0,56	10	Input
	f2	Empirical factor	-	Empirical factor in the overtopping discharge formulation	2,5	10	Input
	hcrest	Crest level of embankment	*m*	Elevation of the crest of the embankment with respect to the ground	4,7-5,39	10	Input
	h	Water level at embankment	*m*	Elevation of the water surface with respect to the ground	4,5-5	10	Input
	γf	Influence factor for rough slope	-	Influence factor for rough slope (0,55 for armour layer)	0,55	10	Input
	γb	Influence factor for oblique waves	-	Influence factor for oblique waves (1 if waves are perpendiculat)	1	10	Input
	γv	Influence factor for vertical wall	-	Influence factor for vertical wall (1 if no vertical walls)	1	10	Input
	f3	Empirical factor	-	Empirical factor in the overtopping discharge formulation	1,3	10	Input
	D	Thickness of slope cover	*m*	Thickness of the cover of the embankment, consisting of concrete blocks	0,07-0,27	11	Output
	Δ	Relative density of concrete	-	Relative density of concrete with respect to water	2,4	11	Input
	F	Stability factor	-	Stability factor for slope cover design (assuming filter below cover)	3,5	11	Input
	b	Empirical exponent	-	Empirical factor for semi-permeable block revetments	0,67	11	Input
	τb,w	Bed shear stress at toe	*N/m2*	Shear stresses by waves at the toe of the embankment	0,4-0,54	12	Output
	ρw	Water density	*kg/m3*	Density of sea water	1030	12	Input
	fw	Friction factor	-	Friction factor associated to bed-shear stresses by waves	0,085	12	Input
	uw,b	Orbital velocity	*m/s2*	Orbital velocity (associated to Hrms) at the sea bottom	0,4-0,5	12	Input
	dn50	Mean grain size of bed	*m*	Mean grain size of sediment particles on the ground	0,0000007	13	Input
	ωm	Mean wave frequency	*rad/s*	Mean wave frequency associated to waves	1,08-1,27	13	Input
